# Using photorespiratory oxygen response to analyse leaf mesophyll resistance

**DOI:** 10.1007/s11120-020-00716-z

**Published:** 2020-02-10

**Authors:** Xinyou Yin, Peter E. L. van der Putten, Daniel Belay, Paul C. Struik

**Affiliations:** 1 Centre for Crop Systems Analysis, Wageningen University & Research, P.O. Box 430, 6700 AK Wageningen, The Netherlands; 2 Selale University, P.O. Box 245, Fiche, Ethiopia

**Keywords:** CO_2_ compensation point, CO_2_ transfer, Internal conductance, O_2_ response, Resistance, Re-assimilation

## Abstract

Classical approaches to estimate mesophyll conductance ignore differences in resistance components for CO_2_ from intercellular air spaces (IAS) and CO_2_ from photorespiration (*F*) and respiration (*R*_d_). Consequently, mesophyll conductance apparently becomes sensitive to (photo)respiration relative to net photosynthesis, (*F* + *R*_d_)/*A*. This sensitivity depends on several hard-to-measure anatomical properties of mesophyll cells. We developed a method to estimate the parameter *m* (0 ≤ *m* ≤ 1) that lumps these anatomical properties, using gas exchange and chlorophyll fluorescence measurements where (*F* + *R*_d_)/*A* ratios vary. This method was applied to tomato and rice leaves measured at five O_2_ levels. The estimated *m* was 0.3 for tomato but 0.0 for rice, suggesting that classical approaches implying *m* = 0 work well for rice. The mesophyll conductance taking the *m* factor into account still responded to irradiance, CO_2_, and O_2_ levels, similar to response patterns of stomatal conductance to these variables. Largely due to different *m* values, the fraction of (photo)respired CO_2_ being refixed within mesophyll cells was lower in tomato than in rice. But that was compensated for by the higher fraction via IAS, making the total re-fixation similar for both species. These results, agreeing with CO_2_ compensation point estimates, support our method of effectively analysing mesophyll resistance.

**Electronic supplementary material:**

The online version of this article (10.1007/s11120-020-00716-z) contains supplementary material, which is available to authorized users.

## Introduction

Quantifying the CO_2_ diffusion inside leaves of C_3_ plants is important in both physiological and ecological contexts. Physiologists assess leaf photosynthetic efficiency and capacity, and both of them depend on how CO_2_ from the atmosphere travel to the chloroplast stroma and how much CO_2_ released by respiration and photorespiration [“(photo)respired CO_2_” hereafter] can be refixed by Rubisco (Busch et al. 2013; von Caemmerer 2013). Ecologists often project the impact of global land CO_2_ fertilization (Sun et al. 2014). The model of Farquhar, von Caemmerer and Berry (1980; “the FvCB model” hereafter), which is widely used as a component for this projection, requires the CO_2_ level at carboxylation sites of Rubisco (*C*_c_) as its input. The drawdown of *C*_c_, relative to the CO_2_ level in the ambient air (*C*_a_), depends not only on stomatal conductance for CO_2_ transfer (*g*_sc_) but also on mesophyll conductance (*g*_m_), such that (von Caemmerer and Evans 1991):

1 $${C}_{\mathrm{c}}={C}_{\mathrm{i}}-A/{g}_{\mathrm{m}}$$

where *C*_i_ is the intercellular air space (IAS) CO_2_ level and *A* is the net photosynthesis rate.

The FvCB model calculates *A* as the minimum of the Rubisco activity limited rate (*A*_c_) and electron transport-limited rate (*A*_j_) of photosynthesis, and Sharkey (1985) added a third limitation, accounting for the rate set by triose phosphate utilization (*A*_p_) (see Supplementary Text S1). Equation (1) has been combined with the FvCB model to estimate *g*_m_ from combined data of gas exchange and chlorophyll fluorescence measurements on photosystem II (PSII) electron transport efficiency *Φ*_2_ (Harley et al. 1992; Yin and Struik 2009). The most commonly used method to estimate *g*_m_ is the ‘variable J method’ (Harley et al. 1992), derived from the *A*_j_ part of the FvCB model, using measurements that have to include photorespiratory conditions (Laisk et al. 2006; see Supplementary Text S2). Equation (1) has also been used to estimate *g*_m_ from online carbon isotope discrimination measurements (e.g. Evans et al. 1994; Tazoe et al. 2011; Barbour et al. 2016a) or oxygen isotope techniques (Barbour et al. 2016b).

Equation (1), as the classical *g*_m_ model, treats the (photo)respired CO_2_ in the same way as it treats the CO_2_ flux that comes from the IAS. Mesophyll resistance (the inverse of mesophyll conductance) consists of components imposed by IAS, cell wall, plasmalemma, cytosol, chloroplast envelope and stroma (Evans et al. 2009; Terashima et al. 2011). Unlike the CO_2_ from the IAS, the (photo)respired CO_2_, mainly coming from the mitochondria, does not need to cross the cell wall and plasmalemma, and thus experiences a different resistance. For this reason, Tholen et al. (2012) developed an Equation for the drawdown of *C*_c_, relative to *C*_i_:

2 $${C}_{\mathrm{c}}={ C}_{\mathrm{i}}-A\left({r}_{\mathrm{w}\mathrm{p}}+{r}_{\mathrm{c}\mathrm{h}}\right)-(F+{R}_{\mathrm{d}}){r}_{\mathrm{c}\mathrm{h}}$$

where *F* and *R*_d_ are CO_2_ fluxes from photorespiration and respiration, respectively, *r*_wp_ is the combined cell wall and plasma membrane resistance, and *r*_ch_ is the chloroplast envelope and stroma resistance (*r*_ch_). Combining Eqs. (1) and (2) results in *g*_m_ = 1/[*r*_wp_ + *r*_ch_ + *r*_ch_(*F* + *R*_d_)/*A*]. Tholen et al. (2012) concluded that mesophyll conductance, as defined by Eq. (1), is influenced by the ratio of (photo)respired CO_2_ release to net CO_2_ uptake, (*F* + *R*_d_)/*A*, thereby resulting in an apparent sensitivity of mesophyll conductance to [CO_2_] and [O_2_]. As this sensitivity does not imply a change in intrinsic diffusion properties, *g*_m_ as defined by Eq. (1) is an apparent parameter. We shall call it the apparent mesophyll conductance (*g*_m,app_). In developing their model, Tholen et al. (2012) assumed a negligible IAS and cytosol resistance, but Eq. (2) still holds if the IAS resistance is lumped into *r*_wp_, and part of cytosol resistance is lumped into *r*_wp_, and the remaining part is lumped into *r*_ch_ (Berghuijs et al. 2015). If *r*_wp_ and *r*_ch_ both represent physical resistances, the total mesophyll diffusion resistance (*r*_m,dif_) is *r*_wp_ + *r*_ch_, and the model of Tholen et al. can be rewritten as

2a $${g}_{\mathrm{m},\mathrm{a}\mathrm{p}\mathrm{p}}= \frac{1}{{r}_{\mathrm{m},\mathrm{d}\mathrm{i}\mathrm{f}}[1+\omega (F+{R}_{\mathrm{d}})/A]}$$

where *ω* is the fraction of *r*_ch_ in *r*_m,dif_.

However, the relative position of mitochondria and chloroplasts is underrepresented in the model of Tholen et al. (2012). Considering six scenarios of the arrangement of these organelles, Yin and Struik (2017) derived the model: *g*_m,app_ = 1/{*r*_m,dif_[1 + *ω*(1 − *λk*)(*F* + *R*_d_)/*A*]}, where *λ* is the fraction of mitochondria located in the inner cytosol (i.e. the cytosol area between chloroplasts and vacuole), and *k* is a factor allowing an increase (*k* > 1), no change (*k* = 1), and a decrease (0 ≤ *k* < 1) in the fraction of inner (photo)respired CO_2_, caused by gaps when chloroplasts are not continuously aligned. The gaps largely depend on the anatomical parameter *S*_c_/*S*_m_, the ratio of chloroplast area to the mesophyll area exposed to IAS (Sage and Sage 2009). As (1 − *λk*) is between 0 and 1, the model predicts that the sensitivity of *g*_m,app_ to (*F* + *R*_d_)/*A* is lower than Tholen et al. (2012) initially stated (Yin and Struik 2017). The model of Tholen et al. applies to an extreme case, either where mitochondria are located exclusively in the outer cytosol between plasmalemma and chloroplasts (*λ* = 0) or where (photo)respired CO_2_ are completely mixed in cytosol if cytosol resistance is negligible and there are chloroplast gaps (*k* → 0). In another extreme case where mitochondria are located exclusively in the inner cytosol (*λ* = 1) and chloroplasts cover completely the cell periphery (*k* = 1), the model predicts no sensitivity of *g*_m,app_ to (*F* + *R*_d_)/*A*, and Eq. (1) would work well as *g*_m,app_ becomes *g*_m,dif_ (= 1/*r*_m,dif_). Equation (1) also works when *r*_ch_ is negligible compared to *r*_wp_ (*ω* = 0) as if (photo)respired CO_2_ is released in the same organelle where RuBP carboxylation occurs. Either situation (*λk* = 1 or *ω* = 0) can be approximately represented by leaves where mitochondria lies only in the inner cytosol, intimately behind chloroplasts that form a continuum.

Most likely scenarios are somewhere between the two extremes defined by Eqs. (1) and (2), that is, 0 < *λk* < 1 and 0 < *ω* < 1. All these scenarios result in different fractions of re-assimilation of (photo)respired CO_2_ (Yin and Struik 2017), both within mesophyll cells and via IAS (see Supplementary Text S3). It would be useful if *ω*, *λ* and *k* can be measured. One way to derive *ω* is to use individual resistances that can be calculated from microscopic measurements on leaf anatomy (Evans et al. 1994; Peguero-Pino et al. 2012; Tosen et al. 2012a, b; Tomas et al. 2013; Berghuijs et al. 2015), despite uncertainties in the value of gas diffusion coefficients. Another possible method to estimate *ω* is to first estimate *r*_wp_ from oxygen isotope techniques assuming that the outer limit of carbonic anhydrase activity represents the cytosol immediately adjacent to the cell wall (Barbour 2017). Parameter *λ* can be assessed using electron microscope images for mitochondria distribution (Hatakeyama and Ueno 2016). Most difficult is to measure *k*, which depends on *S*_c_/*S*_m_. However, whether a high *S*_c_/*S*_m_ would make *k* > 1 or < 1 would depend on the *λ* value as well as on cytosol resistance, and such a complex relationship is hard to quantify with a simple resistance model. However, because *ω*, *λ* and *k* lump together co-defining the sensitivity of *g*_m,app_ to (*F* + *R*_d_)/*A*, the model of Yin and Struik (2017) can be rewritten to

3 $${g}_{\mathrm{m},\mathrm{a}\mathrm{p}\mathrm{p}}= \frac{1}{{r}_{\mathrm{m},\mathrm{d}\mathrm{i}\mathrm{f}}[1+m(F+{R}_{\mathrm{d}})/A]}$$

where *m* = *ω* (1 − *λk*). Although Eq. (3) looks the same as Eq. (2a), their underlying intracellular fluxes for CO_2_ gradient and re-assimilation differ (see Supplementary Text S3). Equation (3) may be used for estimating *m* from noninvasive gas exchange measurements where (*F* + *R*_d_)/*A* varies.

Many reports (e.g. Flexas et al. 2007a; Vrábl et al. 2009; Yin et al. 2009; Tazoe et al. 2011) showed that *g*_m,app_ responds to changes in [CO_2_] or irradiance levels. *g*_m,app_ was shown in tobacco to increase when [O_2_] was decreased from 21 to 1% (Tholen et al. 2012). All these responses can be described using a phenomenological equation (Yin et al. 2009). Tholen et al. (2012) explained the O_2_ response and the commonly observed decline of *g*_m,app_ with decreasing CO_2_ below the ambient level (e.g. Flexas et al. 2007a; Vrábl et al. 2009; Yin et al. 2009), based on the earlier introduced sensitivity of *g*_m,app_ to (*F* + *R*_d_)/*A*, because both increasing O_2_ and decreasing *C*_i_ increase (*F* + *R*_d_)/*A*. However, the sensitivity of *g*_m,app_ to (*F* + *R*_d_)/*A* cannot explain the observed response of *g*_m,app_ to irradiances. Moreover, it is unknown whether *g*_m,dif_ would be conserved across irradiance, CO_2_ and O_2_ levels.

In this study, we described a method that explores varying (*F* + *R*_d_)/*A* ratios to analyse mesophyll resistance from combined gas exchange and chlorophyll fluorescence measurements. The varying (*F* + *R*_d_)/*A* ratios were mainly created using five levels of O_2_, on two contrasting species tomato and rice. Using these data, we assessed (i) the value of the *m* factor and whether it differs between species, (ii) whether *g*_m,dif_ responds to [CO_2_], irradiance and [O_2_], and (iii) how the re-assimilation of (photo)respired CO_2_ is affected by the *m* factor.

## Materials and methods

### Experiments and growth conditions

Seeds of tomato and rice were sown, and uniform seedlings were transplanted into pots 2 weeks after sowing, in glasshouse compartments. Pots were filled with soil, and after assessing initial soil nutrient contents, extra nutrients were applied (Table 1). Tomato plants were watered regularly, while rice plants were maintained submerged.

**Table 1:** Growth and measurement conditions during the experiments with tomato and rice

	Tomato (cv. Growdena)	Rice (cv. IR64)
*Growth condition*		
Pot size and soil	10 L, with potting soil	7 L, with sandy soil
Initial nutrients (pot^−1^)	1.0 g N, 1.2 g P_2_O_5_, and 2.1 g K_2_O	0.40 g N
Total additional nutrients (pot^−1^)	0.38 g N, 0.12 g P_2_O_5_, and 0.40 g K_2_O	0.50 g N, 0.50 g P_2_O_5_ and 0.50 g K_2_O
Temperature (day/night, °C)	21.4/17.0	28/23
Relative humidity (%)	ca 65	ca 65
Photoperiod (h d^−1^)	16	12
Supplementary lights on (W m^−2^) ^a^	≤ 150	≤ 400
Supplementary lights off (W m^−2^) ^b^	≥ 250	≥ 500
*Measurement conditions*		
Position of measured leaves (from the bottom)	the 9th layer leaf	the 9th main-culm leaf
*A* − *I*_inc_ curves	*I*_inc_ = 20, 45, 70, 100, 150, 200, 500, 1000, 1500 µmol m^−2^ s^−1^ with *C*_a_ = 380 µmol mol^−1^ at each of O_2_ levels 2%, 10%, 21%, 35% and 50%, or with *C*_a_ = 1000 µmol mol^−1^ and O_2_ = 2%	*I*_inc_ = 45, 70, 100, 150, 200, 500, 1000, 1500 µmol m^−2^ s^−1^ with *C*_a_ = 380 µmol mol^−1^ at each of O_2_ levels 2%, 10%, 21%, 35% and 50%, or with *C*_a_ = 1000 µmol mol^−1^ and O_2_ = 2%
*A − C*_i_ curves	*C*_a_ = 50, 65, 80, 100, 150, 200, 380, 760, 1000, 1500 µmol mol^−1^ with *I*_inc_ = 1000 µmol m^−2^ s^−1^ at each of O_2_ levels 2%, 10%, 21%, 35% and 50%	*C*_a_ = 50, 65, 80, 100, 150, 200, 380, 600, 1000, 1500 µmol mol^−1^ with *I*_inc_ = 1000 µmol m^−2^ s^−1^ at each of O_2_ levels 2%, 10%, 21%, 35% and 50%
Leaf temperature (°C)	25	25
Leaf-to-air vapour pressure difference (kPa)	0.7–1.5	0.7–1.5

^a^Threshold solar incident light outside glasshouse when supplementary lights were switched on;

^b^Threshold solar incident light outside glasshouse when supplementary lights were switched off

About 60% of the radiation incident on the glasshouse was transmitted to the plant level. During daytime supplemental light from 600 W HPS Hortilux Schréder lamps (Monster, NL) was automatically switched on when the incident solar flux dropped below a threshold and off when it exceeded a threshold outside glasshouse. These threshold levels were set different for tomato and rice (Table 1), to mimic growth environments of the two species.

### Simultaneous gas exchange and chlorophyll fluorescence measurements

We used the Li-Cor-6400XT open gas exchange system with an integrated fluorescence head enclosing a 2-cm^2^ area (Li-Cor Inc, Lincoln-NE, USA). Young but fully expanded leaves of four replicated plants from staggered sowings were measured for incident irradiance (*I*_inc_) and *C*_a_ response curves in each species (Table 1).

Curves were measured at five O_2_ concentrations (Table 1). Additional light response curves were obtained at 1000 µmol mol^−1^*C*_a_ and 2% O_2_ to establish nearly nonphotorespiratory conditions for calibration (see later). Gas from a cylinder containing a mixture of O_2_ and N_2_ was humidified and supplied via an overflow tube to the air inlet of the Li-Cor where CO_2_ was blended with the gas, and the IRGA was adjusted for O_2_ composition of the gas mixture according to the manufacturer’s instructions. Based on pre-test measurements, we used 7–8 min for each step of an *A* − *I*_inc_ curve, and 3–4 min for each step of an *A* − *C*_i_ curve, to reach a steady state. All CO_2_ exchange data were corrected for CO_2_ leakage into and out of the leaf cuvette, using measurements on boiled leaves (Flexas et al. 2007b), and then *C*_i_ was re-calculated.

When *A* reached steady state at each light or CO_2_ step, steady-state fluorescence (${F}_{\mathrm{s}}$) was recorded. Maximum fluorescence (${F}_{\mathrm{m}}^{^{\prime}}$) was measured using a 0.8 s light pulse of > 8000 µmol m^−2^ s^−1^, or the multiphase flash with each phase of 300 ms and ramp depth of 40% (Loriaux et al. 2013). The PSII operating efficiency ($\Delta F/{F}_{\mathrm{m}}^{^{\prime}}$) was set as $({F}_{\mathrm{m}}^{^{\prime}}-{F}_{\mathrm{s}})/{F}_{\mathrm{m}}^{^{\prime}}$ (Genty et al. 1989).

### Calibration and pre-determination of *R*_d_ and Rubisco parameters

Setting that ${\Phi }_{2}=\Delta F/{F}_{\mathrm{m}}^{^{\prime}}$, *R*_d_ was estimated as the negative intercept of a linear regression of *A* against (*I*_inc_*Φ*_2_/4) using data of *A* − *I*_inc_ curves within the electron transport-limited range for the nonphotorespiratory condition (Yin et al. 2009, 2011). The slope of the regression yields a calibration factor (*s*), which lumps (1) absorptance by leaf photosynthetic pigments, (2) the factor for excitation partitioning to PSII, (3) basal forms of alternative electron transport, (4) any difference between real efficiency of PSII electron transport (*Φ*_2_) and $\Delta F/{F}_{\mathrm{m}}^{^{\prime}}$, and (5) possibly difference in chloroplast populations sampled by gas exchange and by chlorophyll fluorescence (van der Putten et al. 2018). The electron transport rate *J* can then be obtained as $J=s{I}_{\mathrm{i}\mathrm{n}\mathrm{c}}(\Delta F/{F}_{\mathrm{m}}^{^{\prime}})$ (Yin et al. 2009). Like other calibration methods, this procedure assumes that the calibration factor is the same for photorespiratory and nonphotorespiratory conditions, for which photosynthetic rates differ by a factor of (*C*_c_ − Γ_*_)/(*C*_c_ + 2 Γ_*_) (see Eqs. S1.1 and S1.3 in Supplementary Text S1; but with cautions from recent literature, Busch et al. 2018; Tcherkez and Limami 2019).

The parameter Γ_*_ was calculated as 0.5*O*_2_/*S*_c/o_, where *S*_c/o_ is the relative CO_2_/O_2_ specificity of Rubisco (von Caemmerer et al. 1994). Values from in vitro measurements of Cousins et al. (2010) on *S*_c/o_ (= 3.022 mbar μbar^−1^) and Michaelis–Menten coefficients of Rubisco for CO_2_ (*K*_mC_ = 291 μbar) and for O_2_ (*K*_mO_ = 194 mbar) were taken, assuming that Rubisco kinetic constants are conserved among C_3_ species. This assumption was checked by in vivo estimates of *S*_c/o_ from the lower parts of *A* − *C*_i_ curves of five O_2_ levels (see “Results”).

### Model method

After the above parameters were quantified, we first checked whether *g*_m,dif_ was variable based on the combined data of gas exchange and chlorophyll fluorescence. Using measured *A*, *C*_i_ and a tentative value for *m* across its range (0 ≤ *m* ≤ 1), *g*_m,dif_ was calculated as

4 $${g}_{\mathrm{m},\mathrm{d}\mathrm{i}\mathrm{f}}=\frac{A + m\left(F +{ R}_{\mathrm{d}}\right)}{{C}_{\mathrm{i}} -{ C}_{\mathrm{c}}}$$

where* F* and *C*_c_ can be solved from the *A*_j_ equation of the FvCB model, see Eq. (S1.6) in Supplementary Text S1 and Eq. (S2.1) in Supplementary Text S2, respectively. Equation (4) was derived by Yin and Struik (2017, see their Eq. 19), in analogy to the variable J method of Harley et al. (1992; also see Eq. S2.2 in Supplementary Text S2).

The obtained *g*_m,dif_ responded to a change in both *C*_i_ and irradiance (see “Results”). Explaining these responses would need a separate study; to estimate *m*, here we adopted the generic phenomenological equation of Yin et al. (2009) to describe this response:

5 $${g}_{\mathrm{m},\mathrm{d}\mathrm{i}\mathrm{f}}={g}_{\mathrm{m}\mathrm{o},\mathrm{d}\mathrm{i}\mathrm{f}}+\delta (A+{R}_{\mathrm{d}})/({C}_{\mathrm{c}}-{\Gamma }_{*})$$

where *g*_mo,dif_ and *δ* are parameters. If *δ* = 0, Eq. (5) becomes a constant *g*_m,dif_ mode (= *g*_mo,dif_). Any nonzero *δ* would predict a variable *g*_m,dif_ in response to CO_2_, O_2_ and irradiance levels, and if *g*_mo,dif_ = 0, parameter *δ*, as discussed later, represents the carboxylation: mesophyll resistance ratio. Equation (5) was combined with the FvCB and other equations to solve for *A* (Supplementary Text S1, where reasons for using Eq. 5 are also explained). This results in an equation expressing *A* as a function of *C*_i_ and other variables:

6 $$A=(-b\pm \sqrt{{b}^{2}-4ac})/(2a)$$

where

$$a={x}_{2}+{\Gamma }_{*}\left(1-m\right)+\delta ({C}_{\mathrm{i}}+{x}_{2})$$

$$b=m\left({R}_{\mathrm{d}}{x}_{2}+{\Gamma }_{*}{x}_{1}\right)-\left[{x}_{2}+{\Gamma }_{*}\left(1-m\right)\right]\left({x}_{1}-{R}_{\mathrm{d}}\right)-\left({C}_{\mathrm{i}}+{x}_{2}\right)\left[{g}_{\mathrm{m}\mathrm{o},\mathrm{d}\mathrm{i}\mathrm{f}}\left({x}_{2}+{\Gamma }_{*}\right)+\delta \left({x}_{1}-{R}_{\mathrm{d}}\right)\right]-\delta [{x}_{1}\left({C}_{\mathrm{i}}-{\Gamma }_{*}\right)-{R}_{\mathrm{d}}\left({C}_{\mathrm{i}}+{x}_{2}\right)]$$

$$c=-m\left({R}_{\mathrm{d}}{x}_{2}+{\Gamma }_{*}{x}_{1}\right)\left({x}_{1}-{R}_{\mathrm{d}}\right)+\left[{g}_{\mathrm{m}\mathrm{o},\mathrm{d}\mathrm{i}\mathrm{f}}\left({x}_{2}+{\Gamma }_{*}\right)+\delta \left({x}_{1}-{R}_{\mathrm{d}}\right)\right][{x}_{1}\left({C}_{\mathrm{i}}-{\Gamma }_{*}\right)-{R}_{\mathrm{d}}\left({C}_{\mathrm{i}}+{x}_{2}\right)]$$

where *x*_1_ = *V*_cmax_ (maximum carboxylation activity of Rubisco) and *x*_2_ = *K*_mC_(1 + *O*_2_/*K*_mO_) for the *A*_c_-limited conditions; *x*_1_ = *J*/4 and *x*_2_ = 2Γ_*_ for the *A*_j_-limited conditions, and for the *A*_p_-limited conditions: *x*_1_ = 3*T*_p_ (where *T*_p_ is the rate of triose phosphate export from the chloroplast) and *x*_2_ = *− *(1 + 3*α*) Γ_*_ (where *α* is the fraction of glycolate carbon not returned to the chloroplast).

We found that the $\sqrt{{b}^{2}-4ac}$ term of Eq. (6) should always take the – sign for either *A*_c_- or *A*_j_-limited rate, but the solution for *A*_p_ is mathematically complicated if *α* > 0 (see Supplementary Text S4). Our data showed that *A* often declined with increasing *C*_i_ within high *C*_i_ ranges (see “Results”), suggesting the limitation by triose phosphate utilization with *α* > 0 (Harley and Sharkey 1991). We conducted sensitivity analyses to choose a value of *α* although metabolic flux data (Abadie et al. 2018) suggest that its value might be small. We then used Eq. (6) to estimate four parameters: *m* (0 ≤ *m* ≤ 1), *δ*, *V*_cmax_ and *T*_p_, by a nonlinear fitting to all data of *A* − *C*_i_ and *A* − *I*_inc_ curves of the five O_2_ levels (*g*_mo,dif_ was set to zero, see “Results”). For that, *J*, as defined earlier as$s{I}_{\mathrm{i}\mathrm{n}\mathrm{c}}(\Delta F/{F}_{\mathrm{m}}^{^{\prime}})$, were used as input. Our method assumed that *R*_d_ does not vary with [O_2_], and was based on the expectation that neither *V*_cmax_ nor *T*_p_ varies with [O_2_], as confirmed experimentally for *V*_cmax_ (von Caemmerer et al. 1994). The fitting minimizes the sum of squared differences between estimated and measured *A* values, using the GAUSS method in PROC NLIN (SAS Institute, NC, USA). SAS scripts can be obtained upon request.

Once *A* was calculated from Eq. (6), *C*_c_ could be solved from Eq. (S2.1) in Supplementary Text S2. Then, *g*_m,dif_ was re-calculated from Eq. (4) using the estimated *m* and measured *A* and *C*_i_, where *x*_1_ and *x*_2_ terms were chosen according to whether the modelled *A* was *A*_c_-, *A*_j_- or *A*_p_-limited. This showed *g*_m,dif_ in response to CO_2_, irradiance, and O_2_ levels.

With *g*_m,dif_ and other parameters, we calculated the fraction of (photo)respired CO_2_ being refixed (*f*_refix_), the fraction of (photo)respired CO_2_ being refixed within the mesophyll cells (*f*_refix,cell_), and the fraction of (photo)respired CO_2_ being refixed via IAS (*f*_refix,ias_), using Eqs. (S3.4), (S3.5) and (S3.6), respectively, in Supplementary Text S3. In these equations, *r*_sc_ is the stomatal resistance to CO_2_ diffusion (being 1.6 times measured stomatal resistance to water vapour), and *r*_cx_ is the carboxylation resistance (which is $({C}_{\mathrm{c}}+{x}_{2})/{x}_{1}$, von Caemmerer 2000). As discussed in Supplementary Text S3, these calculations need *ω* and *λk* as inputs. The estimate for *m* was 0.3 for tomato and 0.0 for rice (see “Results”). For tomato, we measured *ω* (0.65) for leaves of the same age in the same cultivar “Growdena” (see Berghuijs et al. 2015) for calculating *λk*, from *m* = *ω* (1 − *λk*). For rice, *λk* was set to 1.0 to agree with the estimate that *m* = 0. In such a case, *ω* is not needed as Eqs. (S3.4) and (S3.5) become simplified as Eqs. (S3.3) and (S3.9) in Supplementary Text S3, respectively.

## Results

**Fig. 1:**
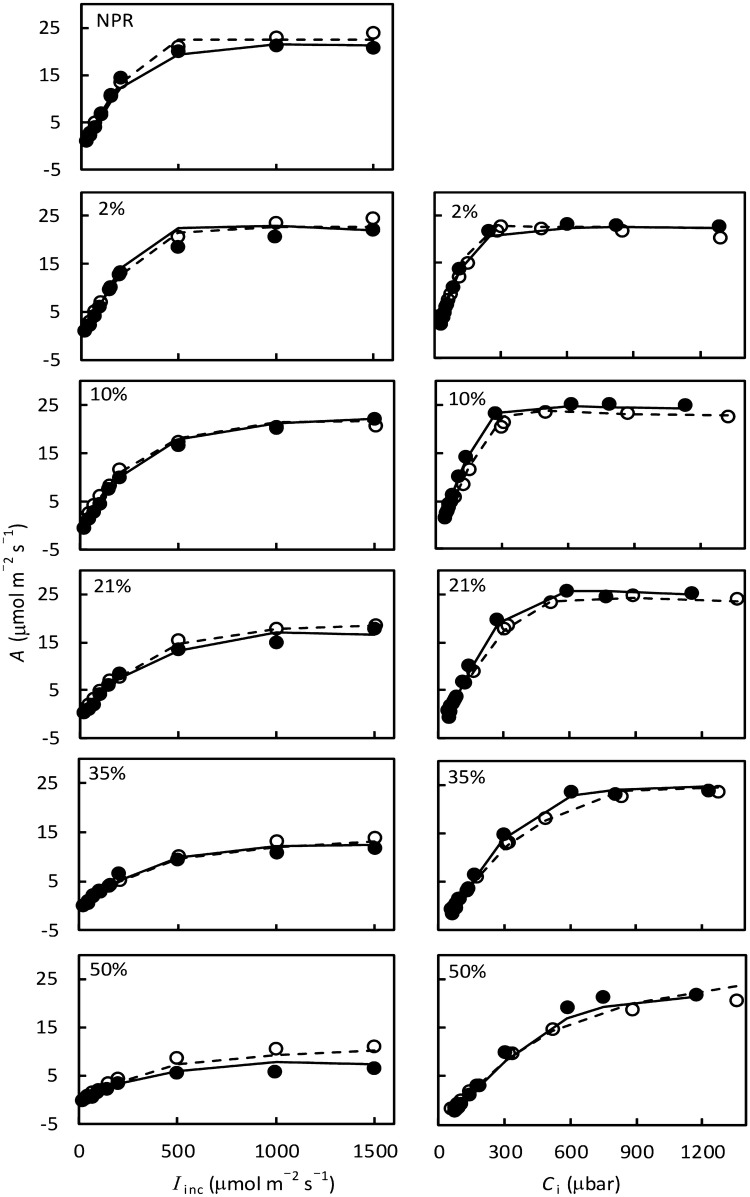
Measured (points) and modelled (curves) net CO_2_ assimilation rate *A* of tomato (filled circle, solid curves) and rice (open circle, dashed curves) as a function of incident irradiance *I*_inc_ (left panels) and of intercellular CO_2_ concentration *C*_i_ (right panels) at different O_2_ percentages as shown in individual panels. Each point represents the mean of four replicated plants. The *A* − *I*_inc_ curve under nonphotorespiratory (NPR) condition was obtained at 2% O_2_ combined with ambient CO_2_ level of 1000 μmol mol^−1^. Curves were drawn from connecting two nearby values calculated by the model

Use of the five O_2_ levels generated diverse shapes of photosynthetic responses to irradiance and CO_2_ levels (Fig. 1). Our model approach, combined with data for *A* (Fig. 1) and for $\Delta F/{F}_{\mathrm{m}}^{^{\prime}}$ (Fig. S1), yielded an estimation of a set of parameters as described below.

### Estimated ***R***_d_ and ***s***

**Fig. 2:**
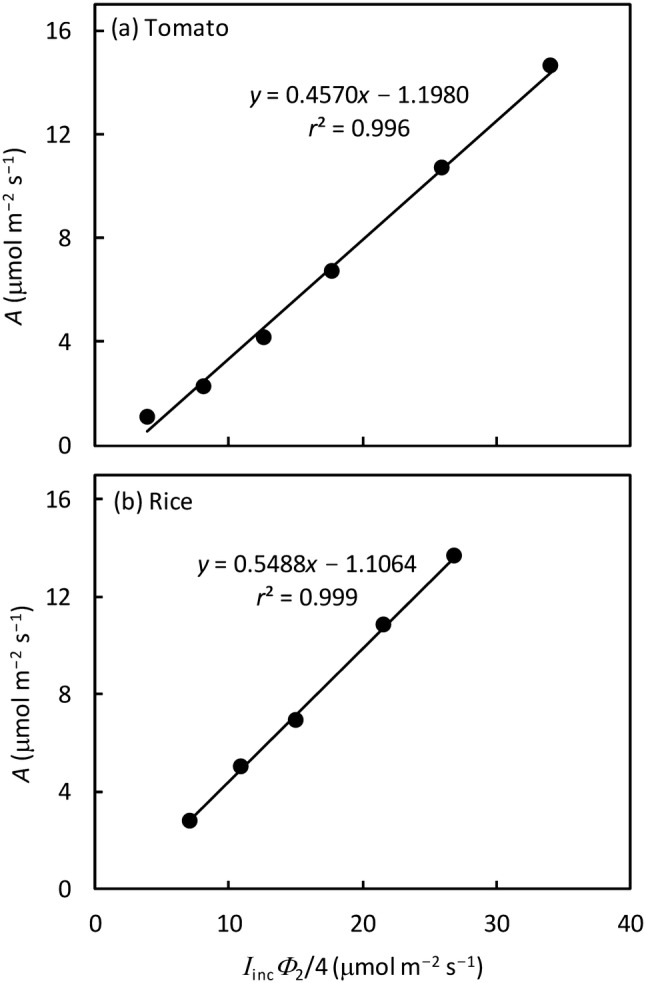
Linear relationship between net CO_2_ assimilation rate *A* and *I*_inc_*Φ*_2_/4, where *Φ*_2_ is set to be $\Delta F/{F}_{\mathrm{m}}^{^{\prime}}$ and *I*_inc_ is ≤ 200 μmol m^−2^ s^−1^ (each point represents the mean of measurements on leaves from four replicated plants), for nonphotorespiratory condition (2% O_2_ combined with *C*_a_ = 1000 μmol mol^−1^). The intercept of regression lines gives an estimate of −*R*_d_ (see Yin et al., 2011), and the slope gives an estimate of the calibration factor *s* for converting $\Delta F/{F}_{\mathrm{m}}^{^{\prime}}$ into the linear electron transport rates (see the text)

Data of *A* − *I*_inc_ curves within the range of *I*_inc_ ≤ 200 µmol m^−2^ s^−1^ showed that the relationship between *A* and (*I*_inc_*Φ*_2_/4) was linear for the conditions with a gas mixture of 2% O_2_ with 1000 μmol mol^−1^*C*_a_ (Fig. 2), where *Φ*_2_ was set to be $\Delta F/{F}_{\mathrm{m}}^{^{\prime}}$. The value of *R*_d_ estimated from this linear relationship was 1.2 (standard error or s.e. 0.1) µmol m^−2^ s^−1^ for tomato and 1.1 (s.e. 0.1) µmol m^−2^ s^−1^ for rice. The slope of the *A* − (*I*_inc_*Φ*_2_/4) linearity (i.e. calibration factor *s*) was 0.4570 (s.e. 0.0076) for tomato and 0.5488 (s.e. 0.0076) for rice. Values of *s* were also re-estimated, together with other parameters, in fitting Eq. (6) to all data; but the re-estimated *s* remained the same, suggesting that we reached a nonphotorespiratory condition using the gas mixture.

**Fig. 3:**
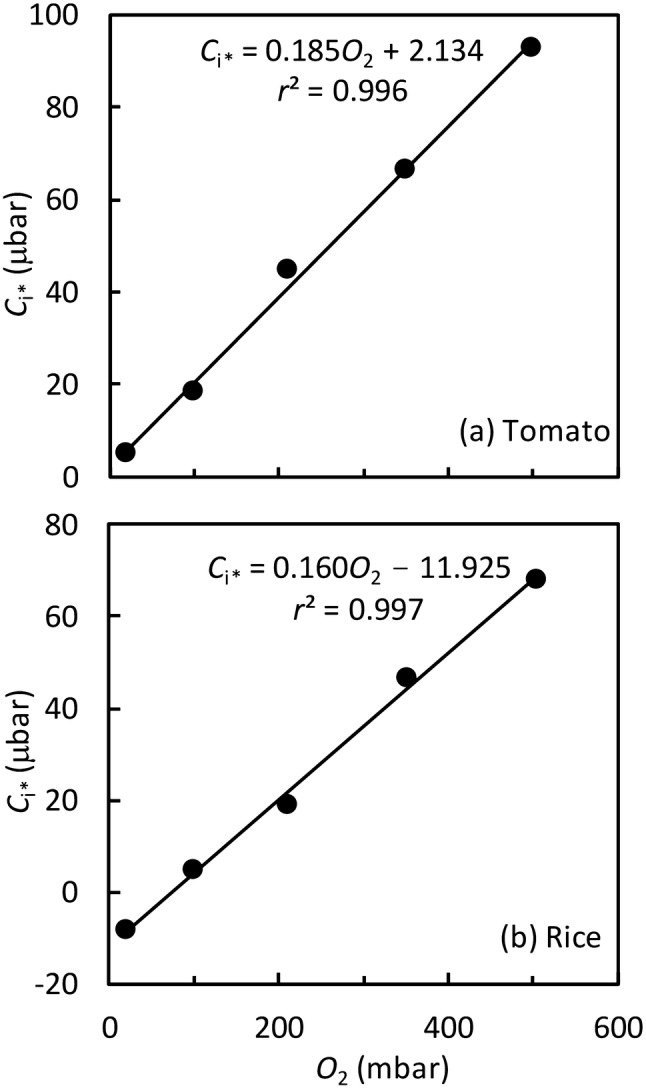
Values of CO_2_ compensation point *C*_i*_ [identified as the intercept at the *C*_i_-axis of the initial strictly linear part of leaf gross CO_2_ assimilation rate (*A* + *R*_d_) versus *C*_i_] plotted as a function of the O_2_ levels, for tomato and rice leaves

The first few data points of the *A* − *C*_i_ curves were linear, and gross leaf photosynthesis values *A* + *R*_d_ were plotted versus *C*_i_ within this linear range. The intercept of this line with the *C*_i_-axis gives the estimate of the *C*_i_-based CO_2_ compensation point, commonly noted as *C*_i*_. The value of *C*_i*_ increased linearly with increasing O_2_ levels (Fig. 3). Half of the reciprocal of this linear slope gives an in vivo estimate of *S*_c/o_, which was 2.71 mbar μbar^−1^ for tomato and 3.13 mbar μbar^−1^ for rice. Using the method of Yin et al. (2009) gave similar in vivo estimates of *S*_c/o_ (results not shown). These values are close to 3.02 mbar μbar^−1^ measured in vitro for wheat by Cousins et al. (2010), confirming that *S*_c/o_ is conserved among C_3_ species. We will use 3.02 mbar μbar^−1^ for further analysis (but see sensitivity analysis later).

### Dependence of *g*_m,dif_ on CO_2_ and irradiance level

**Fig. 4:**
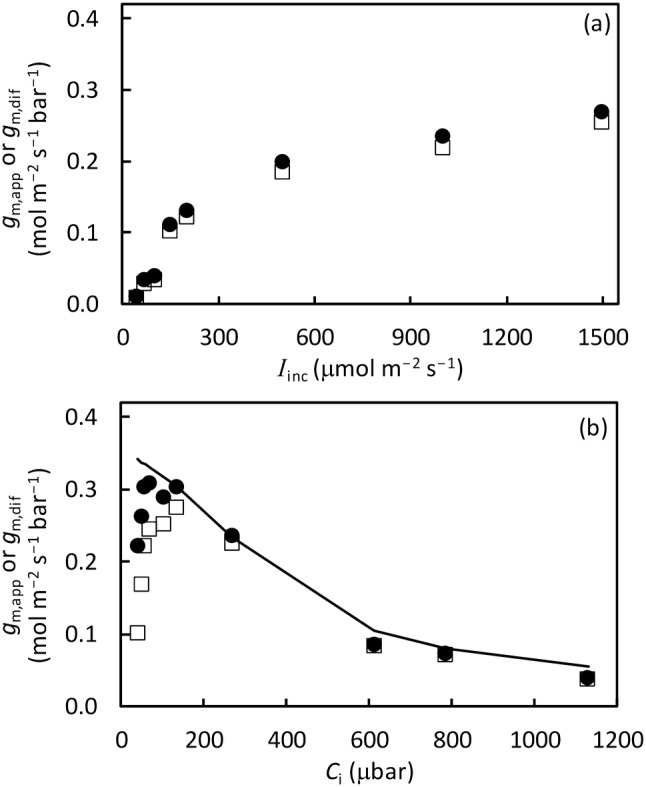
Calculated *g*_m,app_ using the variable J method of Harley et al. (1992) (open square) or *g*_m,dif_ using Eq. (4) where parameter *m* is set to 0.29 (filled circle), as a function of **a** incident irradiance *I*_inc_ or **b** intercellular CO_2_ level *C*_i_, under the condition of 10% O_2_ for tomato leaves. Points were obtained, based on the *A*_j_ part of the FvCB model, using measured *A* and *J* that was derived from chlorophyll fluorescence with the calibration as described in the text. The monotonically descending curve in panel (b) is drawn from values of the modelled *g*_m,dif_ using the full FvCB model of three limited rates

Equation (4) assuming an electron transport limitation, was applied to check the pattern of *g*_m,dif_ across a range of *I*_inc_ and *C*_i_ levels, by setting *m* either to 0 (equivalent to the variable J method of Harley et al. (1992) for *g*_m,app_) or to a value between 0 and 1. A similar response was obtained for various O_2_ levels, except for 2% O_2_. At that oxygen concentration, Eq. (4), like the variable J method, cannot be reliably applied due to insufficient photorespiration (see Supplementary Text S2). Although the obtained *g*_m,dif_ sometimes had unrealistic values largely due to unrealistic values of *C*_c_ (as often occurs when using the variable J method, see Yin and Struik 2009), an overall trend of *g*_m,dif_ in response to *I*_inc_ and to *C*_i_ was obtained. An example of the response is shown in Fig. 4 for the case of 10% O_2_ level for tomato. *g*_m,dif_ increased monotonically with increasing *I*_inc_ (Fig. 4a), and decreased gradually with an increase in *C*_i_ (Fig. 4b). Changing *m* did not change the response pattern, but only the absolute value of *g*_m,dif_, and a nonzero *m* resulted in higher *g*_m,dif_ than the value obtained from setting *m* = 0 (Fig. 4).

### Estimates of parameters *δ*, *m*, *V*_cmax_ and *T*_p_

Equation (6) for describing *A* was applied to estimate *g*_mo,dif_, *δ* and *m*, using data of both *A* − *I*_inc_ and *A* − *C*_i_ curves. The obtained *g*_mo,dif_ did not differ significantly from zero (*p* > 0.05), which is supported by the result that the calculated *g*_m,dif_ by Eq. (4) at low *I*_inc_ was close to zero (Fig. 4a). Also, model fit became worse if *δ* was fixed to zero than if *g*_mo,dif_ was fixed to zero, supporting the variable *g*_m,dif_ mode. Sensitivity analysis with respect to *α* suggested that a change within its relevant range had no impact on the estimates of parameters other than *T*_p_ (see below). We set *g*_mo,dif_ to zero, and *α* to 0.3 (Busch and Sage 2017), in the subsequent analysis.

Equation (6) describes well both *A* − *I*_inc_ and *A* − *C*_i_ curves (Fig. 1), with an overall *R*^2^ being > 0.99 for either species (Table 2). Most of the data points (> 80%) were *A*_j_-limited, indicating that chlorophyll fluorescence signals generally echoed gas exchange data since we calculated *J* from chlorophyll fluorescence measurements as $s{I}_{\mathrm{i}\mathrm{n}\mathrm{c}}(\Delta F/{F}_{\mathrm{m}}^{^{\prime}})$. Only a few points at low *C*_i_ of *A* − *C*_i_ curves or at high *I*_inc_ of *A* − *I*_inc_ curves were *A*_c_-limited, and a few points at high *C*_i_ of *A* − *C*_i_ curves under low O_2_ conditions were *A*_p_-limited. The estimated *m* was ca 0.3 for tomato but was 0.0 for rice (Table 2). The estimated *δ* was also higher for tomato (1.4) than for rice (1.0) (Table 2). Other parameter values were similar for the two species: 113.7 and 111.0 µmol m^−2^ s^−1^ for *V*_cmax_, and 8.3 and 7.8 µmol m^−2^ s^−1^ for *T*_p_, for tomato and rice, respectively.

**Table 2:** Estimates (standard errors in brackets) of two major parameters (*δ* and *m*), and *V*_cmax_ and *T*_p_, from fitting Eq. (6) to irradiance- and CO_2_ response curves of five O_2_ levels for leaves of tomato and rice

Parameter	Unit	Estimates	
		Tomato	Rice
*δ* (a coefficient defining variations in *g*_m,dif_)	–	1.41 (0.09)	1.03 (0.05)
*m* (lumped anatomical-feature parameter)	–	0.29 (0.07)	0.00
*V*_cmax_ (maximum rate of Rubisco activity) ^a^	μmol m^−2^ s^−1^	113.70 (3.51)	111.0 (6.44)
*T*_p_ (rate of triose phosphate utilization) ^b^	μmol m^−2^ s^−1^	8.31 (0.11)	7.81 (0.06)
*R*^2^	–	0.992	0.993

^a^Sensitivity analysis showed that only the estimate of *V*_cmax_ depends on values of *K*_mC_ and *K*_mO_ (see text); here *V*_cmax_ was estimated using *K*_mC_ = 291 μbar and *K*_mO_ = 194 mbar (Cousins et al. 2010)

^b^Sensitivity analysis showed that only the estimate of *T*_p_ depends on the value of *α* (see text); here *T*_p_ was estimated assuming that *α* = 0.3 (Busch and Sage 2017)

### Sensitivity analysis

Given that any uncertainty in estimated *s* and *R*_d_ and in other parameters (*S*_c/o_, *K*_mC_, *K*_mO_ and *α*) may have an impact on the major estimated parameters (*m* and *δ* in this study), we carried out sensitivity analyses. The estimation of *δ* and *m* was very sensitive to *s* and *S*_c/o_, and less sensitive to *R*_d_ (Fig. S2), but virtually insensitive to *K*_mC_, *K*_mO_ and *α* (results not shown). Both *δ* and *m* decreased monotonically with increasing *s* (Fig. S2a). The estimate of *δ* decreased with increasing *S*_c/o_, whereas that of *m* changed in an opposite direction (Fig. S2b). The obtained response of *δ* (the parameter in Eq. (5) on mesophyll conductance) to both *S*_c/o_ and *s* is expected in the same way as *g*_m,app_ responds to these parameters (Harley et al. 1992). The opposite response of *m* to *S*_c/o_ and *s* is probably because photorespiration, i.e. the *F* term in Eq. (3), which is relevant to determining *m*, has an opposite response to *S*_c/o_ and *s*. As *R*_d_ has the same effect as the *F* term has (see Eq. 3), the estimated *m* decreased with increasing *R*_d_, whereas *δ* changed in an opposite direction (Fig. S2c). As expected, any sensitivity to *K*_mC_ and *K*_mO_ occurred with the estimated *V*_cmax_, whereas a sensitivity to *α* occurred with *T*_p_ (results not shown).

### Calculated fractions for re-assimilation of (photo)respired CO_2_

**Fig. 5:**
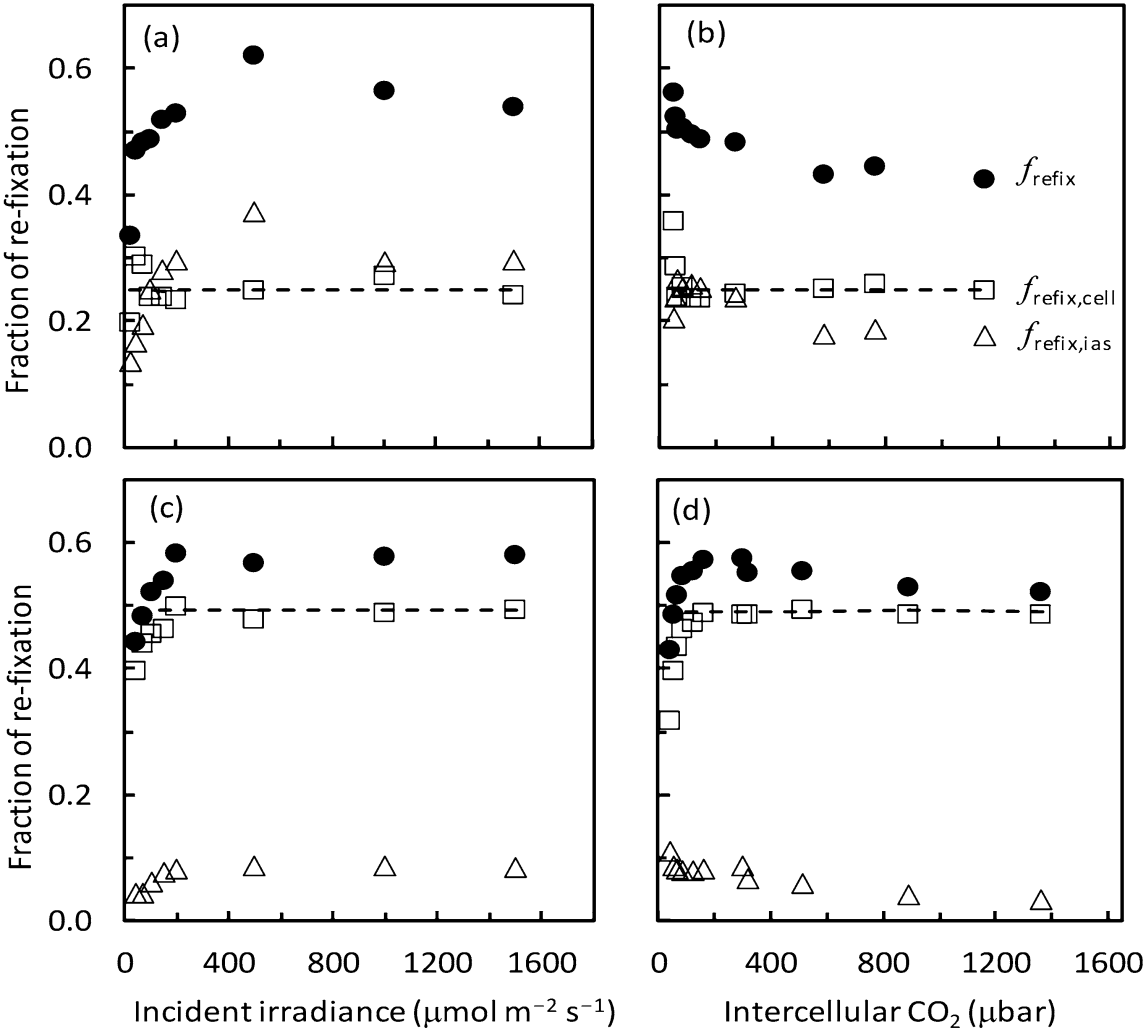
Calculated fractions of total re-assimilation (filled circle, *f*_refix_), of re-assimilation within mesophyll cells (open square, *f*_refix,cell_), and of re-assimilation via the intercellular air spaces (open triangle, *f*_refix,ias_) at different incident irradiance (**a**, **c**) or intercellular CO_2_ (**b**, **d**) levels, in leaves of tomato (**a**, **b**) and rice (**c**, **d**), when the O_2_ level was 21%. The horizontal dashed line represents the calculated *f*_refix,cell_ using the model predicted *A* values. In the calculation for tomato, we used the value of *ω* (the proportion of *r*_ch_ in total *r*_m,dif_) of 0.65 that we measured, as reported by Berghuijs et al. (2015, see the text)

The calculated fractions of (photo)respired CO_2_ being refixed, using Eqs. (S3.4–S3.6) in Supplementary Text S3, are shown in Fig. 5, using the result at 21% O_2_ as the example. The trends were similar for O_2_ levels above 2%. Except for very low *I*_inc_ or *C*_i_ levels, the refixed fractions were quite consistent over a wide range of conditions. *f*_refix,cell_ was lower in tomato (0.25) than in rice (0.49) (Fig. 5), largely due to the fact that the estimated *m* was 0.3 for tomato but 0.0 for rice (Table 2). In contrast, *f*_refix,ias_ was higher in tomato than in rice. As a result, the total re-fixation fraction *f*_refix_ was comparable for the two species, i.e. up to ca 0.6.

### Responses of stomatal and mesophyll conductance to O_2_

**Fig. 6:**
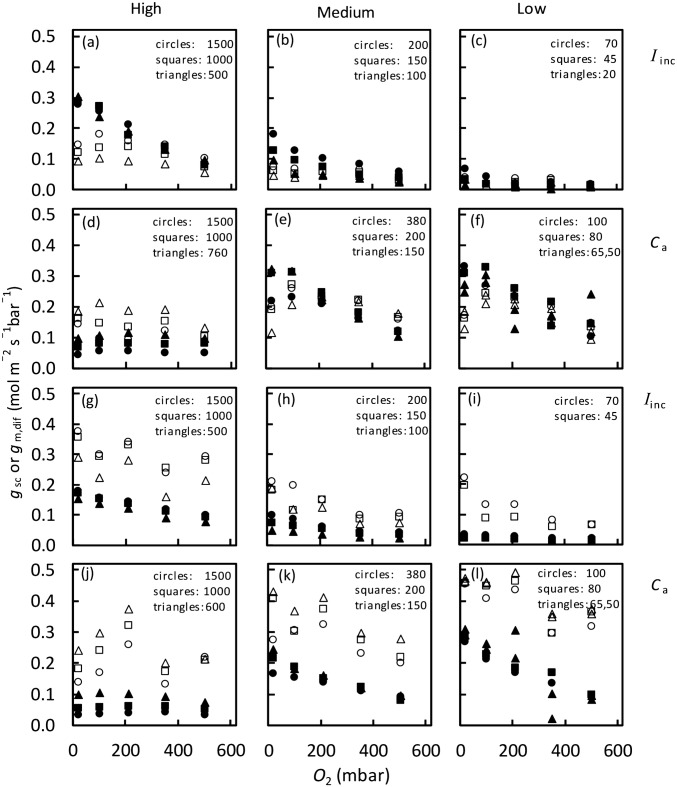
Stomatal conductance for CO_2_ diffusion *g*_sc_ (open symbols) and mesophyll conductance *g*_m,dif_ (closed symbols) of tomato (**a**–**f**) and rice (**g**–**l**) leaves in response to O_2_ level, at high (left panels), medium (middle panels) and low (right panels) *I*_inc_ levels (**a**–**c**, **g**–**i**) or *C*_a_ levels (**d**–**f**, **j**–**l**). Values of *I*_inc_ or *C*_a_ are shown at each corresponding panels, where units of *I*_inc_ and *C*_a_ are μmol m^−2^ s^−1^ and μmol mol^−1^, respectively

Except for a few cases, *g*_sc_ generally decreased with increasing [O_2_], and was lower in tomato than in rice (Fig. 6). The calculated value of *g*_m,dif_ also decreased with increasing [O_2_], except for very high CO_2_ conditions which lowered *g*_m,dif_ to the extent that the O_2_ response of *g*_m,dif_ was no longer significant (Fig. 6d,j). *g*_m,dif_ was higher in tomato than in rice.

## Discussion

### Analysing mesophyll resistance

Compared with the CO_2_ flux coming from IAS, (photo)respired CO_2_ experiences different resistances. This suggests the need to dissect *r*_m,dif_ into sub-components. Anatomical measurements can partition mesophyll resistance into individual sub-components (Peguero-Pina et al. 2012; Tosens et al. 2012a, b; Tomas et al. 2013; Carriquí et al. 2019). The calculation of these sub-components relies on many assumed diffusion or permeability coefficients that are uncertain (Berghuijs et al. 2015). Furthermore, this approach does not quantify the effect of the arrangement of mitochondria and chloroplasts on the intracellular CO_2_ diffusion.

In line with anatomical measurements, Eq. (2) dissects *r*_m,dif_ into two sub-components *r*_wp_ and *r*_ch_ (Tholen et al. 2012). Oxygen isotope techniques may estimate *r*_wp_ based on certain assumptions (Barbour 2017), but so far have been explored to separate *r*_wp_ and *r*_ch_ within the framework of the classical *g*_m_ model, Eq. (1) (Barbour et al. 2016b). Equations (1) and (2) both underrepresent the intracellular arrangements of organelles. In contrast, the model of Yin and Struik (2017), Eq. (3), has a factor lumping (i) the *r*_ch_:*r*_m,dif_ ratio (*ω*), (ii) the fraction of (photo)respired CO_2_ that are released in the inner cytosol (*λ*), and (iii) *k*, the factor for the change in *λ* as a result of the chloroplast gaps. The factor *k* is particularly hard to assess. Since *ω*, *λ* and *k* lump as such that *m* = *ω* (1 − *λk*), Eq. (3) provides an approach by exploring nondestructive gas exchange and chlorophyll fluorescence measurements under different levels of O_2_ that created large variations in photorespiration. Instead of estimating individual resistances, our nonlinear fitting approach estimates *r*_m,dif_ as a whole, as well as the *m* factor. Common nonlinear procedures, typically by fitting *A* − *C*_i_ curves, estimate four or even more parameters of the FvCB model, such as *V*_cmax_, *J*, *T*_p_, and *g*_m_ (e.g. Sharkey et al. 2007). In our method, *J* was measured from chlorophyll fluorescence. Despite a wide range of O_2_ levels exploited, we restricted the number of estimated parameters to four from Eq. (6)-based nonlinear fitting. All four were reliably estimated for both species with small standard errors (Table 2).

Our approach is still a simplified representation of complex diffusion pathways. Some respiratory flux may originate in the chloroplasts, in cytosol, and in the heterotrophic tissues such as epidermis, vasculature, and bundle sheath (Tcherkez et al. 2017). These components of *R*_d_ could be incorporated as additional terms into the *C*_i_–*C*_c_ gradient equation, Eq. (S1.7) in Supplementary Text S1. However, they are ignored here as fractions of these components in *R*_d_ are generally unknown. There may also be some activity of phospho*enol*pyruvate carboxylase (*V*_pepc_) in cytosol (Douthe et al. 2012; Abadie and Tcherkez 2019), which would counteract the effect of (*F* + *R*_d_) on *g*_m,app_. But our procedure of estimating *R*_d_ may have accounted for this, i.e. the estimated *R*_d_ represents the net rate of true *R*_d_ minus *V*_pepc_. Tholen et al. (2012) showed that small amounts of *V*_pepc_ have little impact on *g*_m,app_.

### Variation of *g*_m,dif_ with CO_2_, irradiance and O_2_ levels

Reports using chlorophyll fluorescence data consistently showed that *g*_m,app_ initially increases and then decreases with increasing *C*_i_ and increases monotonically with increasing *I*_inc_ (e.g. Flexas et al. 2007a; Yin et al. 2009). Similar results for *g*_m,app_ in response to *C*_i_ (Vrábl et al. 2009; Tazoe et al. 2011) and to *I*_inc_ (Douthe et al. 2012) were sometimes reported, using the carbon isotope discrimination method. No change in anatomical arrangements was observed that could explain the variable *g*_m,app_ (Carriquí et al. 2019). Gu and Sun (2014) showed that the reported response of *g*_m,app_ to a change in CO_2_ or in *I*_inc_ may be due to the artefact of errors in experimental measurements. Although resolving experimental uncertainties is urgently needed, consistent variations of *g*_m,app_ cannot be ascribed only to experimental errors because responses due to random errors would be irregular and inconsistent among various reports. Théroux-Rancourt and Gilbert (2017) demonstrated that changing patterns of light penetration within the leaf 3D-structure leads to different contributions of each cell layer to bulk-leaf mesophyll conductance, resulting in an apparent response of the bulk-leaf *g*_m,app_ to light intensity. However, their theory cannot explain the response of *g*_m,app_ to *C*_i_.

Most results using the variable J method of Harley et al. (1992) showed that within a low *C*_i_ range, *g*_m,app_ typically decreases with decreasing *C*_i_ (e.g. Flexas et al. 2007a; Vrábl et al. 2009; Yin et al. 2009; Fig. 4b). Tholen et al. (2012) also noted a decrease of *g*_m,app_ with increasing O_2_ level. Tholen et al. (2012) explained these responses to *C*_i_ and O_2_ as a consequence of the fact that *g*_m,app_ as an apparent parameter decreases with an increase in the (*F* + *R*_d_)/*A* ratio. If this is the only explanation of variable *g*_m,app_, one would expect that *g*_m,dif_ would be independent of *C*_i_ because Eq. (4) for *g*_m,dif_ already accounts for the (*F* + *R*_d_)/*A* ratio. However, *g*_m,dif_ still declined with decreasing *C*_i_ within its low range, albeit to a lesser extent (Fig. 4b). In fact, within the low *C*_i_ range, *A* is limited by Rubisco activity; so, as noted by Yin et al. (2009), using the variable J method or Eq. (4) assuming an electron transport limitation results in an artefactual decline of estimated *g*_m_ because the occurrence of additional alternative electron transport is wrongly attributed to the mesophyll diffusional limitation. This assertion was supported by the result that the decrease of *g*_m,dif_ with decreasing *C*_i_ within the low *C*_i_ range was no longer obtained once *g*_m,dif_ was calculated from the full FvCB model (Fig. 4b). This suggests that the decrease of *g*_m,app_ with decreasing *C*_i_ is explained more by the occurrence of alternative electron transport than by the theory of Tholen et al. Anyway, the theory does not explain the decreases of *g*_m,app_ with increasing *C*_i_ within its high range, or with decreasing *I*_inc_, or with lowering temperature as reported previously (Bernacchi et al. 2002; Warren and Dreyer 2006; Yamori et al. 2006; Scafaro et al. 2011; Evans and von Caemmerer 2013).

We found that *g*_m,dif_ increased with *I*_inc_ (Fig. 4a). This increase continued within the high *I*_inc_ range, where some additional alternative electron transport is also expected, suggesting that the increase of *g*_m,dif_ with *I*_inc_ overrode any artefactual decline caused by alternative electron fluxes. Also, *g*_m,dif_ decreased with increasing O_2_ (Fig. 6), in the same direction as the O_2_ response of *g*_m,app_ reported by Tholen et al. (2012). Literature on O_2_ responses of diffusional conductance is scarce (Farquhar and Wong 1984; Buckley et al. 2003). Our data showed that both *g*_sc_ and *g*_m,dif_ generally declined with increasing O_2_. So, *g*_m,dif_ is variable, in response to *C*_i_, *I*_inc_, and O_2_, in a similar pattern as *g*_sc_ responds to these variables (e.g. Morison and Gifford 1983; Farquhar and Wong 1984; Buckley et al. 2003).

The identified variable *g*_m,dif_ was based on the assumption that *m* (= *ω* (1-*λk*)) is constant, independent of short-term changes (within 3–8 min) in irradiance or [CO_2_]. This is supported by Carriquí et al. (2019), who reported that anatomical parameters determining *ω* and *k* hardly vary with short-term changes in irradiance or [CO_2_]. Chloroplasts and mitochondria in some plants may move under varying light, but they always colocalize (Islam et al. 2009), suggesting that *λ* also hardly varies. We are unable to find evidences supporting quantitative changes that *m* or its components must have to obtain invariable *g*_m,dif_ with irradiance, [CO_2_] and [O_2_].

*g*_m,dif_ defined here is still a bulk-leaf trait. Like bulk-leaf *g*_m,app_, it may not represent intrinsic transport properties. Also, our result on the variable *g*_m,dif_ is subject to experimental confirmation by other methods. If proven true, future studies are needed to examine if the variable *g*_m,dif_ can emerge from fluxes and concentrations across the real 3D-structure of leaves, as well as in relation to membrane permeability and other properties. Here we only describe the response from bulk-leaf equations themselves. *g*_m,dif_ can be formulated from Eq. (4) and *A* = *V* − *F* − *R*_d_ (where *V* is the carboxylation rate) as

7 $${g}_{\mathrm{m},\mathrm{d}\mathrm{i}\mathrm{f}}=\frac{V-(1-m)(F+{R}_{\mathrm{d}})}{{C}_{\mathrm{i}}(1-{C}_{\mathrm{c}}:{C}_{\mathrm{i}})}$$

When *I*_inc_ increases, only the numerator increases significantly; so Eq. (7) predicts that *g*_m,dif_ increases with increasing *I*_inc_. If the CO_2_ gradient from *C*_i_ and *C*_c_ is regulated such that the *C*_c_:*C*_i_ ratio is roughly constant for a given O_2_ level (results not shown), Eq. (7) also predicts that *g*_m,dif_ will decrease monotonically with *C*_i_ because according to the FvCB model, the *V* increment per *C*_i_ increment decreases with increasing *C*_i_. Finally, the *F* term increases when O_2_ increases; as a result, Eq. (7) predicts that *g*_m,dif_ decreased with increasing O_2_ (Fig. 6).

### Interpretation of the model and estimated parameter values

Our method is based on Eq. (3), the equation summarized by Yin and Struik (2017) from considering six possible scenarios for the intracellular organelle arrangement. Recently, Ubierna et al. (2019) came up with the same model but formulated *g*_m,app_ in a Michaelis–Menten-like equation, i.e. *g*_m,app_ = *A·g*_m,dif_/[*A* + *m*(*F* + *R*_d_)] (see their Eq. 15; note that *g*_m,app_ was written as *g*_m_ in their notations). The maximum value of *g*_m,app_ is *g*_m,dif_, while the Michaelis–Menten constant “*K*_m_” is *m*(*F* + *R*_d_). For the case of tomato where *m* = 0.3 and *R*_d_ = 1.2, the “*K*_m_” occurs at *A* ≈ 2.0 μmol m^−2^ s^−1^ for the ambient O_2_ condition. This suggests that *g*_m,app_ and *g*_m,dif_ only differ significantly when *A* is low, which our results (Fig. 4) confirmed.

In view of the variation of *g*_m,dif_ shown in Fig. 4, we adopted Eq. (5), which accommodates either constant or variable *g*_m,dif_ in relation to *C*_i_, *I*_inc_ and O_2_ levels. Although the equation is phenomenological and has an a priori assumption that *g*_m,dif_ grows with relative carboxylation and the estimates of its parameters are expectedly sensitive to the pre-input values of *s*, *S*_c/o_ and *R*_d_ (Fig. S2), the model generated useful insights.

Our results supported no constant *g*_m,dif_, but a variable *g*_m,dif_ with parameter *δ* being 1.0 for rice and 1.4 for tomato (Table 2). Equation (5) with *g*_mo,dif_ = 0 for our variable *g*_m,dif_ mode can be rewritten to ${r}_{\mathrm{m},\mathrm{d}\mathrm{i}\mathrm{f}}=({C}_{\mathrm{c}}-{\Gamma }_{*})/[\delta \left(A+{R}_{\mathrm{d}}\right)]$. As $\left(A+{R}_{\mathrm{d}}\right)$ can be calculated from the FvCB model as $({C}_{\mathrm{c}}-{\Gamma }_{*}){x}_{1}/\left({C}_{\mathrm{c}}+{x}_{2}\right)$, the above equation becomes ${r}_{\mathrm{m},\mathrm{d}\mathrm{i}\mathrm{f}}=({C}_{\mathrm{c}}+{x}_{2})/\left(\delta {x}_{1}\right)$. As $({C}_{\mathrm{c}}+{x}_{2})/{x}_{1}$ is defined as carboxylation resistance *r*_cx_ (von Caemmerer 2000), it follows that

8 $$\delta ={{r}_{\mathrm{c}\mathrm{x}}/r}_{\mathrm{m},\mathrm{d}\mathrm{i}\mathrm{f}.}$$

Thus, parameter *δ* of Eq. (5) has a meaning, representing the carboxylation: mesophyll resistance ratio. Our estimates for *δ* (Table 2) suggest that ${{r}_{\mathrm{c}\mathrm{x}}\, \mathrm{a}\mathrm{n}\mathrm{d}\, r}_{\mathrm{m},\mathrm{d}\mathrm{i}\mathrm{f}}$ had similar values in rice leaves, whereas *r*_cx_ was ca 40% higher than *r*_m,dif_ in tomato leaves.

Our estimate of the factor *m* was ca 0.3 for tomato and 0.0 for rice (Table 2). Thus, using Eq. (1), which is the special case of the generalized model when *m* = 0, actually suits for rice leaves but does not work for tomato leaves when (*F* + *R*_d_)/*A* is high. As stated in Introduction, the classical model works well if mitochondria are located exclusively in the inner cytosol (*λ* = 1) and chloroplasts cover fully the mesophyll periphery that *k* = 1. Sage and Sage (2009) and Busch et al. (2013) showed that compared with other species, in rice leaves, there are stromules that effectively extend chloroplast coverage of the cell periphery and mitochondria locate in the cell interior and are intimately associated with chloroplasts/stromules. These features engender such a structure as if (photo)respired CO_2_ is released in the same compartment where RuBP carboxylation occurs. This is the case when Eq. (1) works well. Therefore, our results with curve-fitting to gas exchange data actually agree with anatomical differences between species.

Such differences are also shown in the fractions of re-fixation of (photo)respired CO_2_ calculated from resistance components (Fig. 5). With the distinct anatomical feature of rice leaves, (photo)respired CO_2_, if to exit mesophyll cells, will have to travel via the stroma, thereby maximizing the re-fixation of (photo)respired CO_2_ within the cell. Therefore, rice had higher values of *f*_refix,cell_ than tomato (Fig. 5). For a given set of resistance values, the organelle arrangements as in rice leaves that make the highest *f*_refix,cell_ can result in low *f*_refix,ias_ (see Supplementary Text S3). Moreover, in line with the observation of Ouyang et al. (2017) on rice ‘IR64′, the cultivar we used, rice had high stomatal conductance, compared with tomato (Fig. 6). A low *g*_sc_ would make (photo)respired CO_2_ more difficult to exit into the atmosphere via IAS. This also contributed to higher values of *f*_refix,ias_ in tomato than in rice (Fig. 5). As a result, the two species had similar values (up to 60%) of the total re-fixation, *f*_refix_. The calculated *f*_refix,ias_ and *f*_refix_ varied with *I*_inc_ or *C*_i_ levels (Fig. 5), because resistance components *r*_sc_ and *r*_cx_ varied with these variables. The calculated *f*_refix,cell_ was more constant (Fig. 5). Substituting Eq. (8) into Eq. (S3.5) in Supplementary Text S3 gives

9 $${f}_{\mathrm{r}\mathrm{e}\mathrm{f}\mathrm{i}\mathrm{x},\mathrm{c}\mathrm{e}\mathrm{l}\mathrm{l}}=\frac{(1-\omega )(\delta +\omega \lambda k)}{\left(1+\delta \right)[\delta -\omega \lambda k(\delta +\omega -1)]}$$

As all terms are constant, Eq. (9) describes why *f*_refix,cell_ stayed invariant. Using isotope mass spectrometry and gas exchange measurements, Busch et al. (2013) determined *f*_refix,cell_, *f*_refix,ias_ and *f*_refix_, being 0.29, 0.22, and 0.51 for rice under ambient CO_2_ and high light conditions. Our estimates for rice somewhat differed from their values for comparable conditions (Fig. 5d).

The difference in the value of factor *m* between the species also has implications on values of *C*_i*_ and the relationship between *C*_i*_ and Γ_*_. *C*_i*_ was lower in rice than in tomato at a given O_2_ level (Fig. 3), and *C*_i*_ at the lowest O_2_ in rice was even negative (Fig. 3b). A negative *C*_i*_ could be due to measurement noises, uncertainties in assuming constant *R*_d_, and the influence of varying amounts of *V*_pepc_ (see earlier discussions). However, for the case where *m* = 0, it can be seen from Eq. (1) that *C*_i*_ = Γ_*_ – *R*_d_/*g*_m_ (von Caemmerer et al. 1994); so, *C*_i*_ is always lower than Γ_*_. This agrees with our linear relation for rice in Fig. 3b (where the term 0.16*O*_2_ can be considered as Γ_*_, given that Γ_*_ = 0.5*O*_2_/*S*_c/o_). Setting the intercept of this relation equal to –*R*_d_/*g*_m_ and knowing that *R*_d_ = 1.064 µmol m^−2^ s^−1^ (Fig. 2b), *g*_m_ for rice can be solved as ca 0.1 mol m^−2^ s^−1^ bar^−1^, comparable with its value calculated in the other way for low CO_2_ conditions (Fig. 6l). So, a negative *C*_i*_ for low O_2_ conditions could actually represent biological realities, i.e. high intracellular re-fixation of both respired CO_2_ and photorespired CO_2_ sufficed to (over)compensate for photorespiratory losses. In contrast, for cases where *m* ≥ 0, the relation between *C*_i*_ and Γ_*_ can be formulated from Eq. (S1.7) in Supplementary Text S1 as

10 $${C}_{\mathrm{i}\mathrm{*}}={ \Gamma }_{\mathrm{*}}-[\left(1-m\right){R}_{\mathrm{d}}-mF]{/g}_{\mathrm{m},\mathrm{d}\mathrm{i}\mathrm{f}}$$

Equation (10) means that *C*_i*_ is no longer necessarily lower than Γ_*_ (see also Tholen et al. 2012), depending on relative values of (1–*m*)*R*_d_ versus *mF*. Our result in Fig. 3a suggests that *C*_i*_ is 2.134 μbar higher than Γ_*_ for tomato. Thus, different *m* values estimated by curve-fitting for the two species are supported by the *C*_i*_ vs Γ_*_ relationships in Fig. 3, suggesting that our approach is internally consistent.

## Electronic supplementary material

Below is the link to the electronic supplementary material.

Supplementary file1 (DOCX 344 kb)
