# Supplementary material for: Using photorespiratory oxygen response to analyse leaf mesophyll resistance
Source: Photosynth Res. 2020 Feb 10;144(1):85–99. doi: 10.1007/s11120-020-00716-z (PMC7113236; doi:10.1007/s11120-020-00716-z)
Supplement: Supplementary file 1 — Supplementary file1 (DOCX 344 kb) [file 11120_2020_716_MOESM1_ESM.docx]

The following Electronic Supplementary Material is available for the article entitled “**Using photorespiratory oxygen-response to analyse leaf mesophyll resistance”,** by Xinyou Yin, Peter E.L. van der Putten, Daniel Belay & Paul C. Struik:

**Fig. S1** The PSII operating photochemical efficiency of tomato and rice as a function of incident irradiance and of intercellular CO_2_ concentration at different O_2_ percentages

**Fig. S2** Sensitivity of the estimated values of parameters *δ* and *m* in response to changes in calibration factor *s*, Rubisco specificity *S*_c/o_, and day respiration *R*_d_.

**Text S1** The FvCB model and other equations combined to derive eqn (6)

**Text S2** On the need for photorespiratory conditions when using the variable J method to estimate *g*_m_

**Text S3** Using resistance components to calculate the refixation fraction of (photo)respired CO_2_

**Text S4** Considerations on the mathematical solution to eqn (6) for the *A*_p_-limited rate

**Fig. S1** Measured PSII operating photochemical efficiency $(\Delta F/F_{m}^{'})$ of tomato (●) and rice (○) as a function of incident irradiance (*I*_inc_) and of intercellular CO_2_ concentration (*C*_i_) at different O_2_ percentages as shown in individual panels. Each point represents the mean of four replicated plants. Thecurve under nonphotorespiratory (NPR) condition was obtained at 2% O_2_ combined with ambient CO_2_ level of 1000 μmol mol^-1^.

**Fig. S2.** Sensitivity of the estimated values of parameters *δ* (○ for tomato; Δ for rice) and *m* (□ for tomato) in response to changes in (a) calibration factor *s*, (b) Rubisco specificity *S*_c/o_, and (c) day respiration *R*_d_. The estimated *m* was always zero for rice (see the text), and is therefore not shown in this figure.

**Text S1 The FvCB model and other equations combined to derive eqn (6)**

*Individual equations combined to derive eqn (6)*

The FvCB model (Farquhar et al. 1980; extended by Sharkey 1985) calculates the rate of net CO_2_ assimilation rate *A*, from the minimum of Rubisco-limited carboxylation rate (*V*_c_), electron transport-limited rate (*V*_j_), and triose phosphate utilisation (TPU)-limited rate (*V*_p_). Its mathematical expression is:

$A=\left\{ \begin{aligned} \left( 1-\frac{\Gamma_{*}}{C_{c}} \right)\min\left( V_{c},V_{j} \right)-R_{d}, &{\mathrm{if} C}_{c}\leq(1+3\alpha)\Gamma_{*} \\ \left( 1-\frac{\Gamma_{*}}{C_{c}} \right)min(V_{c},V_{j},V_{p})-R_{d}, &{\mathrm{if} C}_{c}>(1+3\alpha)\Gamma_{*} \end{aligned} \right.$ (S1.1)

The three carboxylation rates are expressed as:

$V_{c}=\frac{C_{c}\cdot V_{\mathrm{cmax}}}{C_{c}+K_{\mathrm{mC}}(1+O/K_{\mathrm{mO}})}$ (S1.2)

$V_{j}=\frac{C_{c}\cdot(J/4)}{C_{c}+2\Gamma_{*}}$ (S1.3)

$V_{p}=\frac{C_{c}{\cdot(3T}_{p})}{C_{c}-(1+3\alpha)\Gamma_{*}}$ (S1.4)

and the three equations for net CO_2_-assimilation rate can be collectively expressed as:

$A=\frac{{(C}_{c}-\Gamma_{*})x_{1}}{C_{c}+x_{2}}-R_{d}$ (S1.5)

The symbols in these equations are all defined in the main text. Recently, Busch et al. (2018) modified eqn (S1.3) and eqn (S1.4) by including carbon and electron requirements for nitrogen assimilation via the photorespiratory pathway. This modified model is not chosen for our analysis as it has more parameters that are difficult to estimate.

The term **_*_/*C*_c_ in eqn (S1.1) represents the photorespiration to carboxylation ratio (Farquhar et al. 1980); the photorespiration *F* can thus be calculated as:

$F=\frac{\Gamma_{*}x_{1}}{C_{c}+x_{2}}$ (S1.6)

The *C*_i_-*C*_c_ gradient in the model of Yin & Struik (2017) is given as [see their eqn (13)]:

$C_{c}=C_{i}-[A+m\left( F+R_{d} \right)]{/g}_{m,dif}$. (S1.7)

The phenomonological model for describing the variable *g*_m,dif_, proposed as eqn (5) in the main text, is:

$g_{m,dif}=g_{mo,dif}+\delta(A+R_{d})/(C_{c}-\Gamma_{*})$ (S1.8)

Combining the four equations, eqns (S1.5-S1.8), could solve the four unknowns (*A*, *C*_c_, *F* and *g*_m,dif_). Our primary objective was to find the equation for *A* as a function of *C*_i_ so as to estimate the parameter *m* (and *δ*, in the case of the variable *g*_m,dif_ mode), from fitting the equation to the data of measured *A* and *C*_i_. This results in eqn (6) in the main text, with which the fitting also results in an estimation of other two parameters *V*_cmax_ and *T*_p_.

*Reasons for introducing the phenomenological equation*

It is worthy to point out the necessity to introduce eqn (5) in the main text or eqn (S1.8) above to describe the variable *g*_m,dif_. One may argue that the variable *g*_m,dif_ could be handled without introducing any additional equation, but by implementing a procedure that solves, in series, for *g*_m,dif_, *C*_c_, and other parameters (like *V*_cmax_ and *T*_p_). For example, Moualeu-Ngangue et al. (2017) described a solving/fitting procedure, in the context of the variable *g*_m,app_, where three equations were combined in series – (i) they used the equation of Harley et al. (1992) (also see eqn S2.2 in our Supplementary Text S2) to calculate *g*_m,app_, based on chlorophyll fluorescence derived electron transport rate *J*_F_; (ii) they used the equation *C*_c_ = *C*_i_-*A/g*_m,app_ to obtain *C*_c_; and (iii) they used *C*_c_ as input to the FvCB model, i.e. eqn (S1.5) here, to estimate *V*_cmax_, *J* and *T*_p_.

We have a reservation on this type of fitting procedures. First, the first step of applying the Harley’s method implicitly assumes that this method, which is based on the electron transport-limited equation of the FvCB model, also applies to the Rubisco- or TPU-limited ranges of the curves. *g*_m,app_ would be under-estimated if the Harley’s method is wrongly applied to the Rubisco- or TPU-limit ranges. As discussed in the main text, this is in fact the major reason for the commonly reported decrease of *g*_m,app_ with decreasing *C*_i_ in the lower part of *A*-*C*_i_ curves where *A* is limited by Rubisco activity. Second, the measured *A* was used to calculate *C*_c_ in step (ii). The measured *A* contain measurement noises, which, together with measurement noises in *C*_i_ and *J*_F_, could make the calculated *C*_c_ values highly unrealistic. Third, *J* and *J*_F_ are treated being independent in this approach, thereby, infringing that the two have the same physiological meaning. Lastly, the estimates of *g*_m,app_ in this way are not amenable for proper predictions of *A*, because there is a circular logic in which the measured *A* are used both as input and as the response variable in the fitting procedure. In fact, *g*_m,app_ estimated in this way differs from its estimates without using the measured *A* in the procedure, as demonstrated by Yin & Struik (2009) for the case of the constant *g*_m,app_.

Therefore, instead of following a similar procedure of Moualeu-Ngangue et al. (2017) for the context of *g*_m,dif_, we introduced the additional equation, eqn (5) in the main text or eqn (S1.8) above, to accommodate the variable *g*_m,dif_. Despite the phenomenological nature of the equation with an additional parameter (*δ*) to be estimated, such an approach allows *C*_c_ and *g*_m,dif_ to be calculated with the internal consistency, i.e. based on respective equations for the three limiting rates of the FvCB model.

**Text S2** **On the need for photorespiratory conditions when using the variable J method to estimate *g*_m_**

The expression used by the variable J method (Harley et al. 1992) to calculate *g*_m_ was derived by combining *A*_j_-limited rate of the FvCB model in the Supplementary Text S1, with eqn (1) in the main text. That is achieved first from solving eqn (S1.5) for *C*_c_:

$C_{c}=\frac{\Gamma_{*}x_{1}+x_{2}(A+R_{d})}{x_{1}-(A+R_{d})}$ (S2.1)

where *x*_1_= *J*/4, and *x*_2_= 2**_*_. Substituting it into eqn (1) in the main text and then solving for *g*_m_ give the familiar equation as the variable J method of Harley et al. (1992) to calculate *g*_m_:

$g_{m}=\frac{A}{C_{i} -C_{c}}=\frac{A}{C_{i} - \frac{\Gamma_{*}x_{1}+x_{2}(A+R_{d})}{x_{1}-(A+R_{d})}}$. (S2.2)

However, the equation above for *C*_c_ can be derived from eqn (S1.5) only for the conditions in which there is a minimum amount of photorespiration. Under the nonphotorespiratory conditions that one can use **_*_ = 0 to mimic, *x*_2_ = 2**_*_ = 0 and eqn (S1.5) becomes:

$A_{j}=\frac{{(C}_{c})(J/4)}{C_{c}}-R_{d}=\frac{J}{4}-R_{d}$ (S2.3)

From eqn (S2.3) there is no way to find an equation for *C*_c_ like eqn (S2.1). Therefore, the variable J method only works for the conditions where there are sufficient amounts of photorespiration. In other words, the variable J method to estimate mesophyll conductance relies on the re-assimilation of photorespiratory CO_2_ (Laisk et al. 2006). The same argument can equally be applied to the case when triose phosphate utilisation limits photosynthesis, because *x*_2_ [= $-(1+3\alpha)\Gamma_{*}$] is also equal to zero for the nonphotorespiratory conditions.

The argument, however, does not apply to the case when Rubisco activity limits photosynthesis. This is because for the nonphotorespiratory conditions that are usually achieved using low O_2_, *x*_2_ [= $K_{\mathrm{mC}}(1+O/K_{\mathrm{mO}})$] approaches to $K_{\mathrm{mC}}$, and *C*_c_ can still be solved as:

$C_{c}=\frac{\Gamma_{*}V_{\mathrm{cmax}}+K_{\mathrm{mC}}(A+R_{d})}{V_{\mathrm{cmax}}-(A+R_{d})}$ (S2.4)

However, one would need to know *V*_cmax_ and *K*_mC_ to estimate *g*_m_. Similarly, one needs to know *T*_p_ and *α* if the *A*_p_-limited equation is used. These parameters are generally unknown beforehand. In contrast, values of *J*/4 in the variable J method are obtained from chlorophyll fluorescence measurements.

The triose phosphate utilisation limited photosynthesis is often modelled using a simplified equation (with $\alpha$ = 0), i.e., $A_{p}={3T}_{p}-R_{d}$. With this simplified model, *A* is independent of *C*_c_, and *C*_c_ cannot be solved under either photorespiratory or nonphotorespiratory conditions, and therefore, *g*_m_ cannot be estimated under either conditions if this simple model holds.

**Text S3** **Using resistance components to calculate the refixation fraction of (photo)respired CO_2_**

*The total refixation fraction for different scenarios*

It has been shown by Tholen et al. (2012) that the fraction of (photo)respired CO_2_ that is being refixed (*f*_refix_) can be estimated from underlying resistance components. For example, they derived an equation for *f*_refix_ as (their eqn 14):

$f_{\mathrm{refix}}=\left( \frac{r_{\mathrm{ch}}+r_{\mathrm{cx}}}{r_{\mathrm{sc}}+r_{\mathrm{wp}}}+1 \right)^{-1}$ (S3.1)

They showed using this equation that *f*_refix_ decreased with increasing *r*_ch_ : *r*_m,dif_ ratio, *ω* (the open symbols of Fig. S3.1). Yin & Struik (2017) noted that the model of Tholen et al. (2012) actually applies primarily to the scenario where mitochondria lie in the outer cytosol and chloroplasts form a continuum (no gaps between chloroplasts), and re-expressed the above equation as:

$f_{\mathrm{refix}}=\frac{r_{\mathrm{sc}}+r_{\mathrm{wp}}}{r_{\mathrm{sc}}+r_{\mathrm{wp}}+r_{\mathrm{ch}}+r_{\mathrm{cx}}}=\frac{r_{\mathrm{sc}}+{(1-\omega)r}_{m,dif}}{r_{\mathrm{sc}}+r_{m,dif}+r_{\mathrm{cx}}}$ (S3.2)

**Fig. S3.1** Calculated refixing fractions of (photo)respired CO_2_ by the model of Tholen et al. (2012, i.e. eqns S3.1 or S3.2) (○) or by eqn (S3.3) (●), as a function of the *r*_ch_ to total mesophyll resistance ratio. Parameter values for calculation are based on those provided in their Fig. 6 of Tholen et al. (2012): *r*_sc_ = 3.3, *r*_m,dif_ = 1.8, and *r*_cx_ = 7.0 m s bar mol^-1^.

For the scenario where mitochondria lie in the inner cytosol in the absence of chloroplast gaps (the scenario corresponding to the classical *g*_m_ model), Yin & Struik (2017) showed that *f*_refix_ can be estimated by:

$f_{\mathrm{refix}}=\frac{r_{\mathrm{sc}}+r_{\mathrm{wp}}+r_{\mathrm{ch}}}{r_{\mathrm{sc}}+r_{\mathrm{wp}}+r_{\mathrm{ch}}+r_{\mathrm{cx}}}=\frac{r_{\mathrm{sc}}+r_{m,dif}}{r_{\mathrm{sc}}+r_{m,dif}+r_{\mathrm{cx}}}$ (S3.3)

For this scenario, eqn (S3.3) indicates that *f*_refix_ stays invariant for a given set of $r_{\mathrm{sc}},r_{\mathrm{wp}},r_{\mathrm{ch}}, and r_{\mathrm{cx}}$, irrespective of the *r*_ch_ : *r*_m,dif_ ratio (the filled symbols of Fig. S3.1), because the numerator will no longer depend on the *r*_ch_ : *r*_m,dif_ ratio, unlike the numerator of eqn (S3.2) where *r*_wp_ decreases with increasing the *r*_ch_ : *r*_m,dif_ ratio.

For a more general scenario, Yin & Struik (2017) derived an equation that calculates *f*_refix_ as:

$f_{\mathrm{refix}}=\frac{\frac{\lambda k}{r_{\mathrm{cx}}} + \frac{1-\lambda k}{{\omega\cdot r}_{m,dif} + r_{\mathrm{cx}}}}{\frac{\lambda k}{r_{\mathrm{cx}}} + \frac{1-\lambda k}{{\omega\cdot r}_{m,dif} + r_{\mathrm{cx}}} + \frac{\lambda k}{r_{m,dif}+ r_{\mathrm{sc}}} + \frac{1-\lambda k}{{(1-\omega)\cdot r}_{m,dif} +r_{\mathrm{sc}}}}$ (S3.4)

where, as defined in the main text, *λ* is the fraction of mitochondria in the inner cytosol, and *k* is a factor allowing an increase (*k* > 1), no change (*k* = 1), and a decrease (0 ≤ *k* <1) in the fraction of inner (photo)respired CO_2_, caused by gaps when chloroplasts are not continuously aligned. Eqn (S3.2) is the special case of eqn (S3.4) when* k* = 0, while eqn (S3.3) is the special case of eqn (S3.4) when *λk* = 1 or when *ω* = 0.

*The fraction of refixation within mesophyll cells* *and via intercellular air-spaces*

The total refixation comprises of the refixation within mesophyll cells (*f*_refix,cell_) and the refixation via intercellular air-spaces (*f*_refix,ias_) (Busch et al. 2013). Following the same logic as used by Tholen et al. (2012) and Yin & Struik (2017) for deriving *f*_refix_, the equation for calculating *f*_refix,cell_ can be derived as for eqn (S3.4) but without the *r*_sc_ terms:

$f_{refix,cell}=\frac{\frac{\lambda k}{r_{\mathrm{cx}}} + \frac{1-\lambda k}{{\omega\cdot r}_{m,dif} + r_{\mathrm{cx}}}}{\frac{\lambda k}{r_{\mathrm{cx}}} + \frac{1-\lambda k}{{\omega\cdot r}_{m,dif} + r_{\mathrm{cx}}} + \frac{\lambda k}{r_{m,dif}} + \frac{1-\lambda k}{{(1-\omega)\cdot r}_{m,dif}}}$ (S3.5)

while that for *f*_refix,ias_ can be simply as:

$f_{refix,ias}=f_{\mathrm{refix}}-f_{refic,cell}$ (S3.6)

Equations for *f*_refix,cell_ and *f*_refix,ias_ representing the scenario of the Tholen et al. (2012) model can be generated from these as:

$f_{refix,cell}=\frac{{(1-\omega)\cdot r}_{m,dif}}{r_{m,dif} + r_{\mathrm{cx}}}$ (S3.7)

$f_{refix,ias}=\frac{r_{\mathrm{sc}}({\omega\cdot r}_{m,dif}+r_{\mathrm{cx}})}{(r_{\mathrm{sc}}+r_{m,dif}+r_{\mathrm{cx}})(r_{m,dif}+r_{\mathrm{cx}})}$ (S3.8)

Likewise, equations for *f*_refix,cell_ and *f*_refix,ias_ representing the scenario of the classical *g*_m_ model can be generated as:

$f_{refix,cell}=\frac{r_{m,dif}}{r_{m,dif} + r_{\mathrm{cx}}}$ (S3.9)

$f_{refix,ias}=\frac{r_{\mathrm{sc}}r_{\mathrm{cx}}}{(r_{\mathrm{sc}}+r_{m,dif}+r_{\mathrm{cx}})(r_{m,dif}+r_{\mathrm{cx}})}$ (S3.10)

It is clear, from comparing eqn (S3.9) vs eqn (S3.7) and eqn (S3.10) vs eqn (S3.8), that if *r*_sc_, *r*_m,dif_, and *r*_cx_ all stay the same between scenarios, *f*_refix,cell_ is lower, whereas *f*_refix,ias_ is higher, for the scenario of the Tholen et al. model than for the scenario of the classical *g*_m_ model. But the higher *f*_refix,ias_ cannot compensate for the lower *f*_refix,cell_, because the total refixation fraction *f*_refix_ is lower for the scenario of the Tholen et al. model than for the scenario of the classical *g*_m_ model (comparing eqn S3.2 vs eqn S3.3; also see Fig. S3.1). It is also clear from these equations that *f*_refix,cell_ tends to be nil and *f*_refix,ias_ and *f*_refix_ tend to be negligible under high-CO_2_ conditions, because an infinite *C*_c_ would give an infinite carboxylation resistance *r*_cx_ [note that *r*_cx_, as pointed by the main text, is calculated by (*C*_c_+*x*_2_)/*x*_1_].

*Can f_refix_, f_refix,cell_ and f_refix,ias_ be calculated by simpler formulae?*

It can be seen that *r*_sc_, *r*_m,dif_, *r*_cx_, *λk* and *ω* need to be known to calculate *f*_refix_ and *f*_refix,cell_. Our measurements and estimates can only provide *r*_sc_, *r*_m,dif_, *r*_cx_, and *m* [where *m* = *ω*(1-*λk*)], but not the separate estimates of *λk* and *ω*. It would be great if *f*_refix_ and *f*_refix,cell_ can be calculated from *m*, rather than from *λk* and *ω*.

The other form of the Tholen et al. (2012) model, eqn (2a) in the main text, and the extended model, eqn (3) in the main text, differ only in the parameter (* *vs *m*) defining the sensitivity of *g*_m,app_ to (*F*+*R*_d_)/*A*. Similarly, the underlying equations for the *C*_i_-*C*_c_ gradient, i.e. eqn (2) in the main text vs eqn (S1.7) in Text S1, also differ only in the parameters * *vs *m*. These could easily lead to suggest the use of eqn (S3.2), (S3.7) and (S3.8) but by replacing *ω* with *m*, for calculating the refixation fractions for the general scenario:

$f_{\mathrm{refix}}=\frac{r_{\mathrm{sc}}+{(1-m)r}_{m,dif}}{r_{\mathrm{sc}}+r_{m,dif}+r_{\mathrm{cx}}}$ (S3.11)

$f_{refix,cell}=\frac{{(1-m)\cdot r}_{m,dif}}{r_{m,dif} + r_{\mathrm{cx}}}$ (S3.12)

Now we examine to what extent eqn (S3.11) and eqn (S3.12) represent eqn (S3.4) and eqn (S3.5) for calculating *f*_refix_ and *f*_refix,cell_, respectively, in the case of the general scenario.

Our estimated *m* was ca 0.3 for tomato (Table 2). Using the anatomical-measurement approach, Berghuijs et al. (2015) showed that for tomato leaves, * *was ca 0.65, meaning that the factor (1-*λk*) would be ca 0.45 for tomato. For rice, *m* was estimated to be 0.0, meaning that (1-*λk*) was close to zero since *ω* can hardly be zero. As expected, for rice where *m* is set to 0, simple models always work.

However, for the general case, *f*_refix_ calculated by the complete and simple models may approximately agree, but *f*_refix,cell_ calculated by the simple model tended to be higher than that by the complete model (Fig. S3.2, left panel). As a result, *f*_refix,ias_ resulted from the simple model tended to be lower than that calculated by the complete model. The larger was the *ω* value, the higher was the difference in *f*_refix,cell_ or *f*_refix,ias_ calculated by simple and complete models (Fig. S3.2, middle panel). If *ω* is ≤ 0.4, the error from the simplification was generally within ca 5%.

**Fig. S3.2** Calculated *f*_refix_ (circles), *f*_refix,cell_ (squares), and *f*_refix,ias_ (triangles), by the complete equations (eqns S3.4 – S3.6, closed symbols) vs by the simplified equations (eqn S3.11 and eqn S3.12, open symbols), for for three different combinations of input parameter values indicated in the three panels. Resistance values (m s bar mol^-1^) for calculations in the left and middle panels are the same as in Fig. S3.1, i.e. based on those provided in Fig. 6 of Tholen et al. (2012). Note that any variation of *m* has to be within the range that 0 ≤ *m* ≤ *ω*.

These results may suggest that for some cases the above simplification using eqn (S3.11) roughly works if one is interested in estimating the total refixation fraction *f*_refix_ but the simplification does not work for calculating *f*_refix,cell_ and *f*_refix,ias_ (especially if *ω* > 0.4). However, this conclusion for *f*_refix_ may not hold if the relative values of *r*_sc_, *r*_m,dif_, and *r*_cx_ changed exceptionally (see the right panel of Fig. S3.2).

The model of Tholen et al. (2012) can be used to estimate *ω*, the fraction of *r*_ch_ in total mesophyll resistance *r*_m,dif_, if mitochondria lie in the outer cytosol or if the (photo)respired CO_2_ are completely mixed in cytosol. Our results in Fig. S3.2 suggest that one cannot rename *r*_ch_ from *ωr*_m,dif_ to an effective chloroplast resistance *mr*_m,dif_ in order to make the Tholen et al. model as a tool for the general scenario (equivalent to using the simple equations) to estimate the fraction of the effective chloroplast resistance in *r*_m,dif_. This is because, as can be seen from eqn (12) of Yin & Struik (2017) for the general case when considering the gradient between *C*_m_ (the CO_2_ level in cytoplasm) and *C*_c_:

$C_{c}=C_{m}-[V-\lambda k\left( F+R_{d} \right){]r}_{\mathrm{ch}},$ (S3.13)

the resistance component *r*_ch_, or *ωr*_m,dif_, involves the total flux of carboxylation (*V*), of which only the flux (*F*+*R*_d_) is associated with *λk*. In other words, * *and *λk* do not lump together when quantifying the *intracellular* fluxes to make it possible to conveniently replace *ω* with *m* in determining the fraction of the (effective)*r*_ch_ in total mesophyll resistance. This differs from considering the *C*_i_-*C*_c_ gradient, whereby * *and *λk* lump together into the *m* factor (see eqn S1.7). In relation to that, errors from using simplified equations were generally smaller for calculating *f*_refix_ than for calculating *f*_refix,cell_ and *f*_refix,ias_, as shown in the first two panels of Fig. S3.2.

**Text S4** **Considerations on the mathematical solution to eqn (6) for** **the *A*_p_-limited rate**

As stated in the main text, the mathematical solution for *A* in eqn (6) should take the – sign in front of the $\sqrt{b^{2}-4ac}$ term for either *A*_c_- or *A*_j_-limited rate. However, the solution for *A*_p_ is mathematically complicated when the entire *C*_i_ range is considered, because the *x*_2_ term in eqn (S1.5) for *A*_p_ is negative, i.e., $x_{2}=-(1+3\alpha)\Gamma_{*}$. Model simulation suggests that, depending on values of *α*, *g*_mo,dif_ and *δ*, $(b^{2}-4ac)$ for *A*_p_ is sometimes negative within a certain range of *C*_i_ (meaning no real root for *A*_p_ within the range); while for *C*_i_ lower and higher than the thresholds of the range, the $\sqrt{b^{2}-4ac}$ term of eqn (6) should take the – and + signs, respectively. Exact mathematical expressions for the threshold *C*_i_ are hard to solve from eqn (6).

On the other hand, when applying the *A*_p_-limited rate, one should consider the putative transition point, *C*_c,ts_, from the *V*_j_ to the *V*_p_ limitation. The value for *C*_c,ts_ can easily be solved from eqn (S1.3) for *V*_j_ and eqn (S1.4) for *V*_p_ as:

$C_{c,ts}=\frac{24\Gamma_{*}T_{p}+(1+3\alpha)\Gamma_{*}J}{J-12T_{p}}.$ (S4.1a)

However, the value for *C*_c,ts_ obtained from eqn (S4.1a) can be low when *J* is low under low-irradiance conditions (mathematically *C*_c,ts_ is even negative if $J<12T_{p}$). Then the other constraint for the *A*_p_ limited rate in the FvCB model, $C_{c}$ > $(1+3\alpha)\Gamma_{*}$, should apply (Gu et al. 2010; also see eqn S1.1), because $\mathrm{any} C_{c}$ lower than $(1+3\alpha)\Gamma_{*}$ would make *V*_p_ negative and *V*_p_ would always be chosen from min(*V*_c_, *V*_j_, *V*_p_), whereas physiologically the *V*_p_ limitation occurs at high *C*_c_ (Sharkey 1985). Therefore, eqn (S4.1a) is extended to:

$C_{c,ts}=\max\left[ \frac{24\Gamma_{*}T_{p}+\left( 1+3\alpha\right)\Gamma_{*}J}{J-12T_{p}},(1.0001+3\alpha)\Gamma_{*} \right].$ (S4.1b)

(where 1.0001 is used to avoid a possible division by zero, see a later equation, eqn S4.3).

Eqn (S1.7) applied to this transition point gives a *C*_i_-based value:

$C_{i,ts}-[A_{\mathrm{ts}}+m(F_{\mathrm{ts}}+R_{d})]{/g}_{m,dif,ts}=C_{c,ts}.$ (S4.2a)

*A*_ts_ can be substituted by (*V*_ts_ - *F*_ts_ - *R*_d_). Then eqn (S4.2a) becomes:

$C_{i,ts}-[V_{\mathrm{ts}}-(1-m)(F_{\mathrm{ts}}+R_{d})]{/g}_{m,dif,ts}=C_{c,ts}.$ (S4.2b)

Substituting eqn (S1.5) and eqn (S1.6) for the transition point into eqn (S4.2b) gives:

$C_{i,ts}=C_{c,ts}+\left\{ \frac{[C_{c,ts}-(1-m)\Gamma_{*}]3T_{p}}{C_{c,ts}-(1+3\alpha)\Gamma_{*}}-(1-m)R_{d} \right\}{/g}_{m,dif,ts}.$ (S4.3)

where *g*_m,dif,ts_ can be formulated from eqn (5) and eqn (S1.5) as:

$g_{m,dif,ts}=g_{mo,dif}+\delta\frac{3T_{p}}{C_{c,ts}-(1+3\alpha)\Gamma_{*}}$ (S4.4)

Eqn (S4.3), combined with eqns (S4.1b) and (S4.4), was used to identify *C*_i,ts_, above which *A*_p_ is added to the model for fitting. Simulation showed that the identified *C*_i,ts_ was close to the *C*_i_ obtained using a physiological approach from checking whether $\Delta F/F_{m}^{'}$ starts to decline with increasing *C*_i_ (Sharkey et al. 2007). Our data suggest that above *C*_i,ts_, there was always a mathematical solution for *A*_p_, i.e. $(b^{2}-4ac)$ ≥ 0, and the $\sqrt{b^{2}-4ac}$ term for *A*_p_ should take the + sign. Model simulation using various set of parameter values suggests that the – sign would make the calculated *A*_p_ values biologically unrealistic, either being too high, or being too low (lower than the lower limit 3*T*_p_-*R*_d_), or staying invariant for the case *α *> 0.

Integrating all discussed above, the FvCB model used for fitting was:

$A=\left\{ \begin{aligned} \min\left( A_{c},A_{j} \right), &{\mathrm{if} C}_{i}\leq C_{i,ts} \mathrm{or} \left( b^{2}-4ac \right)<0 for A_{p} \\ min(A_{c},A_{j},A_{p}), &{\mathrm{if} C}_{i}>C_{i,ts} \end{aligned} \right.$ (S4.5)

where *C*_i_ should be ≥ *C*_i*_, and solutions for *A*_c_, *A*_j_, and *A*_p_ are eqn (6) in the main text, with the $\sqrt{b^{2}-4ac}$term taking the – sign for *A*_c_ and *A*_j_ but the + sign for *A*_p_.

**References**

Berghuijs, H.N.C.,Yin, X.,Ho, Q.T., van der Putten, P.E.L., Verboven, P., Retta, M.A., Nicolaï, B.M. and Struik, P.C., 2015. Modelling the relationship between CO_2_ assimilation and leaf anatomical properties in tomato leaves. Plant Science 238: 297-311.

Busch, F.A., Sage, R.F. and Farquhar, G.D., 2018. Plants increases CO_2_ uptake by assimilating nitrogen via the photorespiratory pathway. Nature Plants 4: 46-54.

Busch, F.A., Sage, T.L., Cousins, A.B. and Sage, R.F., 2013. C_3_ plants enhance rates of photosynthesis by reassimilating photorespired and respired CO_2_. Plant, Cell and Environment 36: 200-212.

Farquhar, G.D., von Caemmerer, S. and Berry, J.A., 1980. A biochemical model of photosynthetic CO_2_ assimilation in leaves of C_3_ species. Planta 149: 78-90.

Gu, L., Pallardy, S.D., Tu, K., Law, B.E. and Wullschleger, S.D., 2010. Reliable estimation of biochemical parameters from C_3_ leaf photosynthesis-intercellular carbon dioxide response curves. Plant, Cell and Environment 33: 1852-1874.

Harley, P.C., Loreto, F., Di Marco, G. and Sharkey, T.D., 1992. Theoretical considerations when estimating the mesophyll conductance to CO_2_ flux by analysis of the response of photosynthesis to CO_2_. Plant Physiology 98: 1429-1436.

Laisk, A., Eichelmann, H., Oja, V., Rasulov, B. and Rämma, H., 2006. Photosystem II cycle and alternative electron flow in leaves. Plant and Cell Physiology 47: 972-983.

Moualeu-Ngangue, D.P., Chen, T.-W. and Stutzel, H., 2017. A new method to estimate photosynthetic parameters through net assimilation rate-intercellular CO2 concentration (*A*-*C*_i_) curve and chlorophyll fluorescence measurements. New Phytologist 213: 1543-1554.

Sharkey, T.D., 1985. O_2_-insensitive photosynthesis in C_3_ plants: Its occurrence and a possible explanation. Plant Physiology 78: 71-75.

Sharkey, T.D., Bernacchi, C.J., Farquhar, G.D. and Singsaas, E.L., 2007. Fitting photosynthetic carbon dioxide response curves for C_3_ leaves. Plant, Cell and Environment 30: 1035-1040.

Tholen, D., Ethier, G., Genty, B., Pepin, S. and Zhu, X.-G., 2012. Variable mesophyll conductance revisited: theoretical background and experimental implications. Plant, Cell and Environment 35: 2087-2103.

Yin, X. and Struik, P.C., 2009. Theoretical reconsiderations when estimating the mesophyll conductance to CO_2_ diffusion in leaves of C_3_ plants by analysis of combined gas exchange and chlorophyll fluorescence measurements. Plant, Cell and Environment 32: 1513-1524 (corrigendum in PC&E 33: 1595).

Yin, X. and Struik, P.C., 2017. Simple generalisation of a mesophyll resistance model for various intracellular arrangements of chloroplasts and mitochondria in C_3_ leaves. Photosynthesis Research 132: 211-220.
